# Towards a new combination therapy for tuberculosis with next generation benzothiazinones

**DOI:** 10.1002/emmm.201303575

**Published:** 2014-02-05

**Authors:** Vadim Makarov, Benoit Lechartier, Ming Zhang, João Neres, Astrid M Sar, Susanne A Raadsen, Ruben C Hartkoorn, Olga B Ryabova, Anthony Vocat, Laurent A Decosterd, Nicolas Widmer, Thierry Buclin, Wilbert Bitter, Koen Andries, Florence Pojer, Paul J Dyson, Stewart T Cole

**Affiliations:** 1 More Medicines for Tuberculosis (MM4TB) Consortium http://www.mm4tb.org; 2 Bakh Institute of Biochemistry, Russian Academy of ScienceMoscow, Russia; 3 Global Health Institute, Ecole Polytechnique Fédérale de LausanneLausanne, Switzerland; 4 Institute of Chemical Sciences and Engineering, École Polytechnique Fédérale de LausanneLausanne, Switzerland; 5 Department Medical Microbiology and Infection Control, VU University Medical CenterAmsterdam, The Netherlands; 6 Division of Clinical Pharmacology, CHUV, Hôpital BeaumontLausanne, Switzerland; 7 Department of Molecular Microbiology, VU UniversityAmsterdam, The Netherlands; 8 Janssen Infectious DiseasesBeerse, Belgium

**Keywords:** benzothiazinones, combination regimens, DprE1, tuberculosis

## Abstract

The benzothiazinone lead compound, BTZ043, kills *Mycobacterium tuberculosis* by inhibiting the essential flavo-enzyme DprE1, decaprenylphosphoryl-beta-D-ribose 2-epimerase. Here, we synthesized a new series of piperazine-containing benzothiazinones (PBTZ) and show that, like BTZ043, the preclinical candidate PBTZ169 binds covalently to DprE1. The crystal structure of the DprE1-PBTZ169 complex reveals formation of a semimercaptal adduct with Cys387 in the active site and explains the irreversible inactivation of the enzyme. Compared to BTZ043, PBTZ169 has improved potency, safety and efficacy in zebrafish and mouse models of tuberculosis (TB). When combined with other TB drugs, PBTZ169 showed additive activity against *M. tuberculosis in vitro* except with bedaquiline (BDQ) where synergy was observed. A new regimen comprising PBTZ169, BDQ and pyrazinamide was found to be more efficacious than the standard three drug treatment in a murine model of chronic disease. PBTZ169 is thus an attractive drug candidate to treat TB in humans.

**Subject Categories** Microbiology, Virology & Host Pathogen Interaction; Pharmacology & Drug Discovery

## Introduction

Today, tuberculosis (TB) accounts for the annual loss of approximately 1.5 million lives worldwide and the pandemic is exacerbated by poverty, homelessness, synergy with HIV/AIDS and widespread drug resistance (WHO, 2012). While most of the 9 million new TB cases occur in the developing world, the industrialized nations, especially in Europe, are also at increasing risk due to the inexorable spread of drug-resistant disease through global travel and immigration. There are an estimated 650 000 cases of multidrug-resistant TB (MDR-TB), which no longer respond to frontline treatment, and in excess of 60 000 cases of extensively drug-resistant TB (XDR-TB), that are resistant to key second-line drugs in addition, among the world's 12 million prevalent cases of TB (WHO, 2012). Around 2 billion individuals have latent TB and MDR-TB is currently threatening the success of both the TB and HIV control programs worldwide.

The past decade has seen intensive efforts to discover and develop new drugs to treat drug-susceptible-, MDR- and XDR-TB, and new combination regimens are also being devised and tested in clinical trials (Koul *et al*, 2011; Diacon *et al*, 2012). New regimens will most likely employ a combination of repurposed drugs and new chemical entities (NCE) and there is a real likelihood that these regimens may contain none of the drugs previously used in TB treatment (Zumla *et al*, 2013). Repurposed drugs include members of the fluoroquinolone, oxazolidinone, riminophenazine and rifamycin families. Among the most advanced NCE in phase II and phase III clinical trials are the diarylquinoline, bedaquiline or BDQ (Andries *et al*, 2005; Diacon *et al*, 2009); the bicyclic nitroimidazoles, PA-824 (Stover *et al*, 2000; Diacon *et al*, 2012), delamanid or OPC67683 (Matsumoto *et al*, 2006; Gler, 2012); and SQ109, a novel 1,2-ethylenediamine-based analogue (Lee *et al*, 2003; Tahlan *et al*, 2012).

The current TB drug development pipeline also has extensive activity in the early stages (Zumla *et al*, 2013), but there is a gap, corresponding to late preclinical development and phase I clinical trials, that needs filling to ensure continuity of clinical activity and to compensate for the probable attrition among the more advanced candidates. One NCE that is nearing phase I clinical trials is the benzothiazinone, BTZ043 (2-[(2*S*)-2-methyl-1,4-dioxa-8-azaspiro[4.5]dec-8-yl]-8-nitro-6-(trifluoromethyl)-4*H*-1,3-benzothia-zin-4-one). BTZ043 is one of the most potent inhibitors of *Mycobacterium tuberculosis* yet described, displaying nanomolar bactericidal activity both *in vitro* and in *ex vivo* models of TB (Makarov *et al*, 2009). In murine models of acute and chronic TB, BTZ043 showed efficacy approaching that observed with the frontline drugs isoniazid and rifampin although these are far less potent with respect to their *in vitro* minimal inhibitory concentrations (MIC).

**Figure 1:**
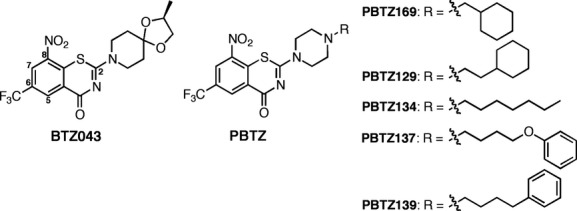
Chemical structures. Structure of BTZ043 and five compounds of the PBTZ family selected for *in vivo* studies, PBTZ169, PBTZ139, PBTZ134, PBTZ137 and PBTZ139.

BTZ043 (Fig 1), a nitroaromatic compound, is active against MDR-clinical isolates of *M. tuberculosis* (Makarov *et al*, 2009; Pasca *et al*, 2010) and targets the essential flavoprotein subunit, DprE1, of decaprenylphosphoryl-beta-D-ribose 2-epimerase. This enzyme produces the sole source of the D-arabinose required for biosynthesis of the key cell wall components arabinogalactan and lipoarabinomannan. BTZ043 serves as a suicide substrate for the reduced form of DprE1 undergoing nitroreduction to yield a nitroso species that specifically attacks the thiol side chain of the active site cysteine residue, Cys387, thereby forming a covalent adduct and irreversibly inactivating the enzyme (Trefzer *et al*, 2010, 2012; Neres *et al*, 2012).

Since the efficacy of BTZ043 in the mouse model of TB was lower than expected from its exceptional potency (MIC - 1 ng/ml; 2.3 nM) and the compound is relatively hydrophobic (log*P *=* *2.84) we suspected that the pharmacologic properties could be improved. Consequently, we have produced a new, enhanced series of benzothiazinones, the PBTZ, by introducing a piperazine group into the scaffold. Here, we present the findings of a comparison of the next-generation preclinical candidate PBTZ169 (Fig 1) with BTZ043, and describe its superior properties and suitability for inclusion in a new combination regimen for the treatment of both drug-susceptible and drug-resistant TB.

## Results

### SAR studies of piperazine-containing benzothiazinone derivatives

During our structure-activity relationship (SAR) analysis of the first generation benzothiazinone (BTZ) compounds we discovered a direct correlation between lipophilicity (log*P*) and antimycobacterial activity (Makarov *et al*, 2009). The aim of the present research was to design and synthesize a new generation of BTZ derivatives, that were sufficiently lipophilic to be highly antimycobacterial but with better pharmacodynamic parameters to increase their *in vivo* efficacy. Based on our prior knowledge of the BTZ scaffold, with its great affinity for DprE1, it was clear that even minor structural changes led to loss of antimicrobial activity. For instance, key constituents are the sulfur and oxygen atoms in the thiazine ring, strong electron acceptor groups (CF_3_, CN, NO_2_ etc.) in position 6, and protons in positions 5 and 7 (Fig 1). The essentiality of the nitro group in position 8 was demonstrated by synthesizing and characterizing many benzothiazinone derivatives with alternative electron withdrawing groups in that position, but these were all inactive (Makarov *et al*, 2009). An explanation for this was provided when the mechanism of action was elucidated and the structure of the BTZ043-DprE1 complex was solved (Trefzer *et al*, 2010, 2012; Neres *et al*, 2012).

Based on our previous studies, only position 2 of the BTZ scaffold remained for derivatization. We designed a new series of substituted 2-piperazino-benzothiazinones (PBTZs), which allowed extensive SAR studies through variation of the piperazine N-4 substituents, which were rapidly synthesized from the parent substituted piperazines. From the SAR studies performed, it soon became evident that hydrophilic groups including alcohols, carboxylic acids and secondary or tertiary amines invariably led to loss of antimycobacterial activity when compared to BTZ043 (Supplementary Table 1).

A second family of compounds, with more hydrophobic substituents on the piperazine ring was then synthesized. In this case, the small hydrophilic and saturated piperazine moiety is “hidden” between two hydrophobic groups: the crucial 1,3-benzothiazin-4-one ring and the N-4 piperazino substituent. A series of aryl-PBTZs, containing substituted phenyl rings or pyridyl moieties was synthesized and found to have low *in vitro* and *in vivo* activity (Supplementary Table 1). Finally, a series of alkyl-PBTZs was synthesized, which had the advantages of improving the aqueous solubility of the resulting compounds through protonation of the tertiary amino nitrogen of the piperazine ring, and the fact that the hydrophobicity of the molecule could be readily modulated through introduction of various alkyl groups (Table 1 and Supplementary Table 1). Details of the general synthetic route may be found in the supporting information and elsewhere (Makarov, 2011).

**Table 1:** Correlation between MIC of selected PBTZ and log*P*

Compound number	Alkyl substitute	MIC *M. tuberculosis* (ng/ml)	logP*
10926013	Methyl	250	1.31
10926021	Ethyl	62	1.64
10926027	Propyl	3.7	2.11
10926172	Butyl	1.9	2.51
11026100	Isobutyl	1.9	2.51
11026142	1-Ethylpropyl	0.37	2.99
11026128	1-*sec*-Butyl	0.37	2.52
11026129, PBTZ129	2-Cyclohexylethyl	0.19	3.52
11026131	1-Methylbutyl	0.19	3.11
11026134, PBTZ134	Heptyl	0.19	3.30
11026137, PBTZ137	4-Phenoxybutyl	1.5	3.35
11026139, PBTZ139	4-Phenylbutyl	0.37	4.05
10926168	Cyclohexyl	0.75	3.09
10926169, PBTZ169	Cyclohexylmethyl	≤0.19	3.20

* Calculated using Hyperchem 7.5 (Hypercube Inc., http://www.hyper.com).

The *in vitro* activities against *M. tuberculosis* H37Rv of the 60 resultant PBTZ compounds were measured by the resazurin reduction microplate assay (REMA). The alkyl-PBTZ series of compounds showed the most potent anti-tuberculosis activity and displayed a strong correlation between MIC and lipophilicity (Table 1 and Supplementary Table 1), based on their respective calculated log*P*s. Selected PBTZ compounds displaying MICs ranging from 0.19 to 0.75 ng/ml against *M. tuberculosis* H37Rv were tested in their free base and hydrochloride salt forms but the MIC values obtained were identical. The *in vivo* efficacy of a range of PBTZ derivatives (PBTZ169, 129, 134, 137, 139) was then tested in the chronic TB mouse model and the results are presented below. The most attractive compound was PBTZ169, which appears more stable than the other derivatives synthesized probably because its cyclohexyl group protects against enzymatic attack by nitroreductases and the methylene linker is sandwiched between two bulky groups.

### Microbiological characterization of PBTZ169 and selected derivatives

The MIC_99_ of PBTZ169 and BTZ043 were determined for a variety of actinobacteria by REMA. Two TB drugs, moxifloxicin (MXF) and rifampicin (RIF) were used for control purposes. PBTZ169 was found to be three- to seven-fold more active than BTZ043 against *M. tuberculosis*, *Mycobacterium bovis* BCG, *Mycobacterium marinum*, *Mycobacterium smegmatis*, *Corynebacterium diphtheriae* and *Corynebacterium glutamicum* (Table 2). PBTZ169 and BTZ043 were not active against atypical mycobacteria like *M. avium* (Makarov *et al*, 2009) nor against *M. abscessus, M. boletti*, *M. massiliense* and *M. vaccae* (Table 2) probably because these mycobacteria possess *dprE1* genes where the codon equivalent to Cys387 of *M. tuberculosis* has been replaced by an Ala or Ser codon (see below). Consistent with previous findings (Makarov *et al*, 2009; Pasca *et al*, 2010), both PBTZ169 and BTZ043 were active against MDR- and XDR-clinical isolates of *M. tuberculosis* (Table 2 and Supplementary Table 2).

**Table 2:** MIC determinations for different Actinobacteria by REMA

	MIC_9__9_ (μg/ml)			
	BTZ043	PBTZ169	RIF	MXF
*C. diphtheriae* DSM 44123	0.06	0.003	0.0008	0.02
*C. glutamicum* ATCC 13032	0.063	0.013	0.004	0.31
*M. abscessus* 2005-0524	>100	>100	6.25	0.78
*M. avium* ATCC 15769	>100	>100	25	0.47
*M. bolletii* 1999-0888	>100	>100	50	0.94
*M. bovis* BCG Pasteur	0.001	0.0006	0.001	0.02
*M. bovis* BCG BN2	>100	>100	0.0008	0.03
*M. marinum* Strain M	0.0007	0.0003	0.39	0.1
*M. massiliense* 2005-0484	>100	>100	25	0.96
*M. smegmatis* mc^2^155	0.001	0.0007	0.5	0.06
*M. smegmatis* GM22	0.031	0.003	1	0.06
*M. smegmatis* MN47	25	6.25	1	0.06
*M. smegmatis* MN84	>100	>100	1	0.06
*M. tuberculosis* H37Rv	0.001	0.0003	0.001	0.03
*M. tuberculosis* NTB1	25	>100	0.001	0.03
*M. tuberculosis* MDR_CHUV	0.002	0.0004	>100	0.03
*M. vaccae* ATCC 15483	>100	>100	100	0.63

To establish whether cross-resistance, indicative of a common target, was observed between BTZ043 and PBTZ169 we tested two BTZ-resistant mutants, both with Cys387Ser missense mutations in DprE1, namely, *M. tuberculosis* NTB1 and *M. bovis* BCG BN2, and the drug-susceptible parent strains. The mutations conferred >10 000-fold increased resistance to both compounds (Table 2). The activities of BTZ043 and PBTZ169 were also compared against three BTZ-resistant mutants of *M. smegmatis*, two of which harbor missense mutations in DprE1 (MN47 Cys394Gly; MN84 Cys394Ser) (Makarov *et al*, 2009) whereas the third overproduces the NfnB nitroreductase (GM22) (Manina *et al*, 2010) that confers partial resistance by inactivating BTZ043. Again the DprE1 mutants displayed cross-resistance to both compounds, but PBTZ169 proved to be far less susceptible than BTZ043 to inactivation by NfnB since their MICs for GM22 were 5 and 50 ng/ml, respectively (Supplementary Fig 1).

### Effect of nitroreduction by NfnB on PBTZ169 and selected derivatives

**Figure 2:**
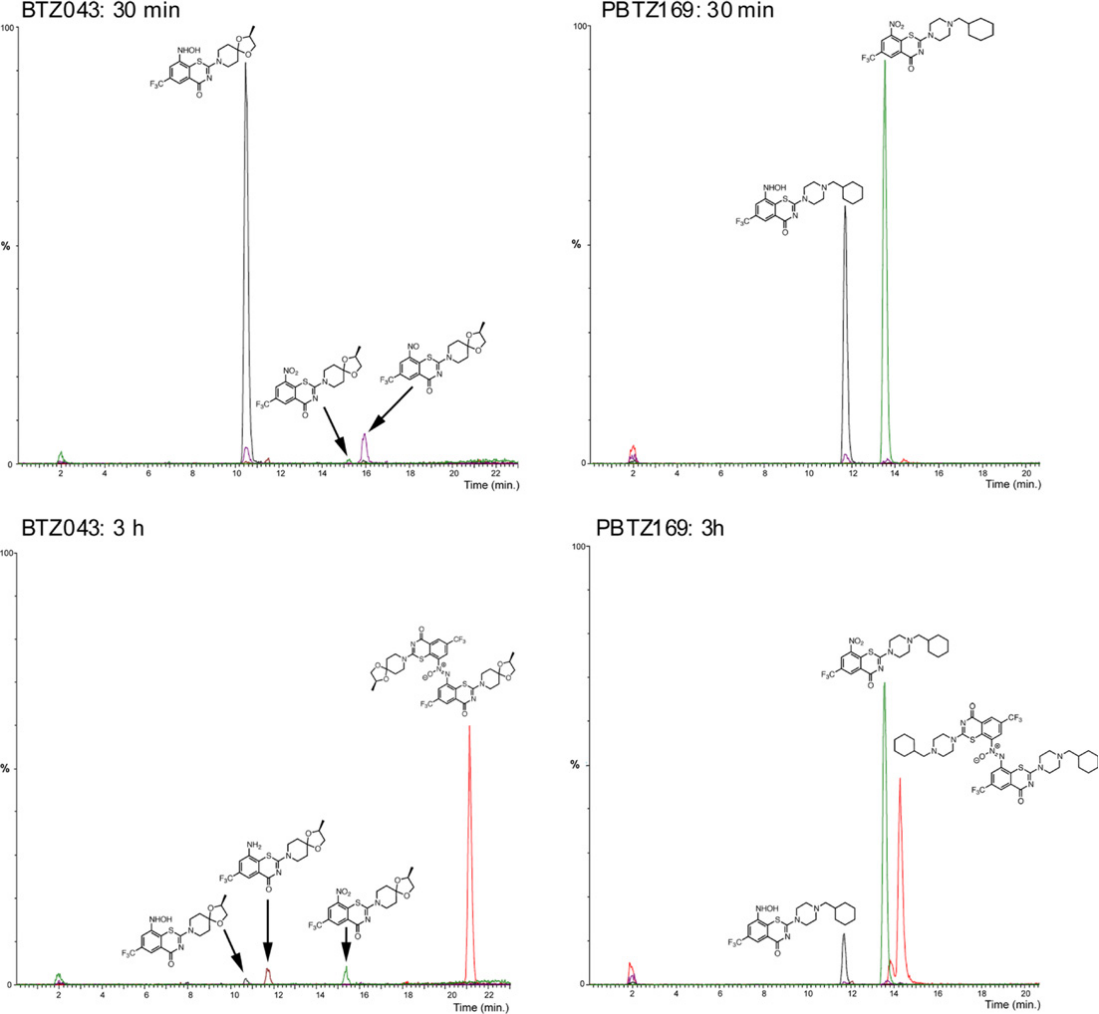
NfnB-mediated reduction of BTZ043 and PBTZ169. Compounds (50 μM) were incubated with NfnB (6 μM) as described in Materials and Methods. Samples (10 μL) were taken at different times during the reaction and directly analyzed by LC-MS (ESI+). Results were analyzed by extracting the ion counts for the species of interest for each compound, namely the protonated forms of the original compound, and its nitroso, hydroxylamino and amino forms, as well as the azoxy adducts.

To confirm that PBTZ169 was indeed more resistant than BTZ043 to nitroreduction by NfnB, both compounds were incubated with purified recombinant *M. smegmatis* NfnB, as described previously (Manina *et al*, 2010), and samples taken periodically for LC-MS analysis. BTZ043 was reduced very quickly and after 30 min had been almost entirely converted to its hydroxylamino analogue and a trace amount of the nitroso intermediate (Fig 2). After 3 h incubation with NfnB, a new species was present with a molecular weight of 814, observed in the LC-MS trace in its doubly-protonated form (m/z = 433). This likely corresponds to the azoxy adduct formed from two nitroso-BTZ043 molecules, as suggested by Tiwari *et al* (Tiwari *et al*, 2013).

The fate of PBTZ169 on incubation with NfnB was quite different from that of BTZ043, as even after 3 h incubation, the major species found in the LC-MS analysis was the original nitro form. As observed with BTZ043, the hydroxylamino and azoxy adducts of PBTZ169 were also present in the assay mixture, but in much smaller amounts. Similar results (data not shown) were obtained for PBTZ129, PBTZ137 and PBTZ139 (Table 1). However, one of the PBTZs tested, PBTZ134, was as susceptible to NfnB-mediated reduction as BTZ043.

### Enzymology: DprE1 inhibition *in vitro*

Inhibition of *M. tuberculosis* DprE1 by PBTZ169 and BTZ043 was monitored by determining the residual enzyme activity following incubation with a range of concentrations of these compounds, using a two step coupled enzyme assay (Neres *et al*, 2012). For this purpose, we expressed and purified *M. tuberculosis* DprE1 (Batt *et al*, 2012). The results showed that full inhibition of DprE1 was obtained following 5 min incubation with 5 μM PBTZ169 whereas 20 μM BTZ043 was required to achieve this (Supplementary Fig 2A). To confirm that PBTZ169 and BTZ043 shared the same mechanism for DprE1 inhibition, namely the formation of a covalent adduct with the active site cysteine, we incubated DprE1 with PBTZ169 in the presence of the FPR (farnesylphosphoryl-ß-D-ribofuranose) substrate. Mass spectrometry analysis of the protein in the reaction mixture clearly showed that the expected semimercaptal adduct was formed between PBTZ169 and DprE1 (Supplementary Fig 2B) (Trefzer *et al*, 2010, 2012; Neres *et al*, 2012).

### Crystal structure of the DprE1-PBTZ169 adduct

**Figure 3:**
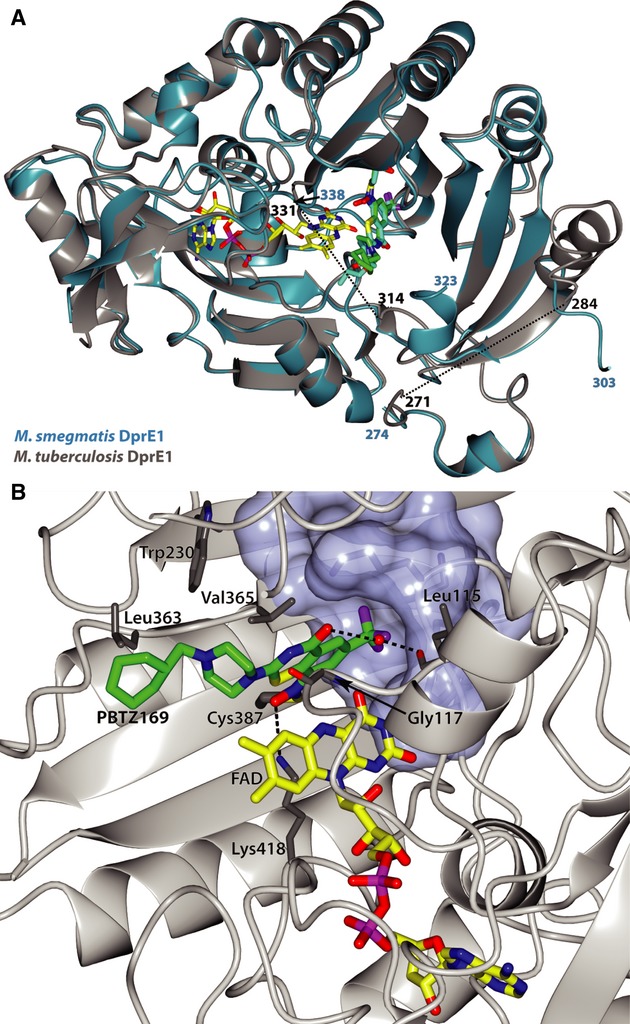
Crystal structure of the M. tuberculosis DprE1-PBTZ169 complex.
Cartoon representation of the *M. tuberculosis* DprE1 structure (grey) in complex with PBTZ169 adduct (green sticks), and its FAD cofactor (yellow sticks), superposed on the *M. smegmatis* DprE1 structure (blue) in complex with BTZ043 and FAD. The location of the disordered regions in the *M. tuberculosis* DprE1 are marked as black dashed lines. Residue numbers are indicated in black for the *M. tuberculosis* structure and in blue for the *M. smegmatis* protein.Close-up view of the *M. tuberculosis* DprE1 active site showing the residues in close contact with PBTZ169. The binding pocket of the CF_3_ group of PBTZ169 is shown as a surface representation.

The structural and mechanistic similarities between BTZ043 and PBTZ169 pointed to similar modes of binding to the active site of DprE1 so, to fully characterize this, we determined the crystal structure of the *M. tuberculosis* DprE1-PBTZ169 adduct using the procedure previously reported for the *M. smegmatis* DprE1-BTZ043 structure (Neres *et al*, 2012). DprE1-PBTZ169 crystals diffracted to 1.9 Å resolution (Supplementary Table 3). The structure (PDB 4NCR) was solved by molecular replacement using the native *M. tuberculosis* DprE1 structure (PDB 4FDP (Batt *et al*, 2012)) as a model. The overall fold of the *M. tuberculosis* and *M. smegmatis* DprE1 enzymes, and the position of the benzothiazinones in the respective active sites are very similar, as shown in Fig 3A.

Closer inspection of the active site of the DprE1-PBTZ169 structure (Fig 3B) shows that the benzothiazinone ring interacts with the same residues in the active site as previously observed in the *M. smegmatis* DprE1-BTZ043 structure. The electron density map does not account fully for the cyclohexyl moiety of PBTZ169 (Supplementary Fig 3A), likely due to its higher flexibility. The cyclohexylmethyl-piperazine moiety is placed between the FAD flavin ring and residues Gly117, Trp230 and Leu363 (Fig 3B), with the side chains of the latter two adopting slightly different conformations when compared with the DprE1-BTZ043 complex structure. A water molecule bridges a hydrogen bond between the carbonyl oxygen of the BTZ ring and the backbone carbonyl of Leu115. Interestingly, after modelling in the PBTZ169 structure and refinement, extra electron density was observed in a pocket lined by PBTZ169 itself and the residues Trp111, Lys134, Phe199, amino acids 226-230, Phe313 and Tyr314. Given its proximity to the active site, and the narrow elongated shape, we hypothesized that this corresponded to bound FPX (farnesylphosphoryl-D-2-keto-*erythro*-pentose), the product obtained by DprE1 oxidizing FPR. We modelled the FPX structure in the mentioned electron density (Supplementary Fig 3B), and verified that except for its sugar moiety, placed in a free region between residues Trp230 and Gln308 with high B factors and essentially no electron density, the phosphate and farnesyl moieties fitted quite well. Therefore, it is highly likely that the polyprenyl chain of the DprE1 substrate is located adjacent to the BTZ binding site.

### *In vitro* ADME/T characterization

The potential cytotoxicity of BTZ043 and PBTZ169 was assessed using the HepG2 human cell line (Supplementary Table 4). PBTZ169 was found to be 10-times less cytotoxic (TD_50_ of 58 μg/ml) compared to BTZ043 (TD_50_ of 5 μg/ml). Both compounds thus have excellent selectivity indices of >10 000. On incubation with human or mouse microsomes, both BTZ043 and PBTZ169 showed medium clearance values (Supplementary Table 4).

### Efficacy of PBTZ169 against *M. marinum* in zebrafish

**Figure 4:**
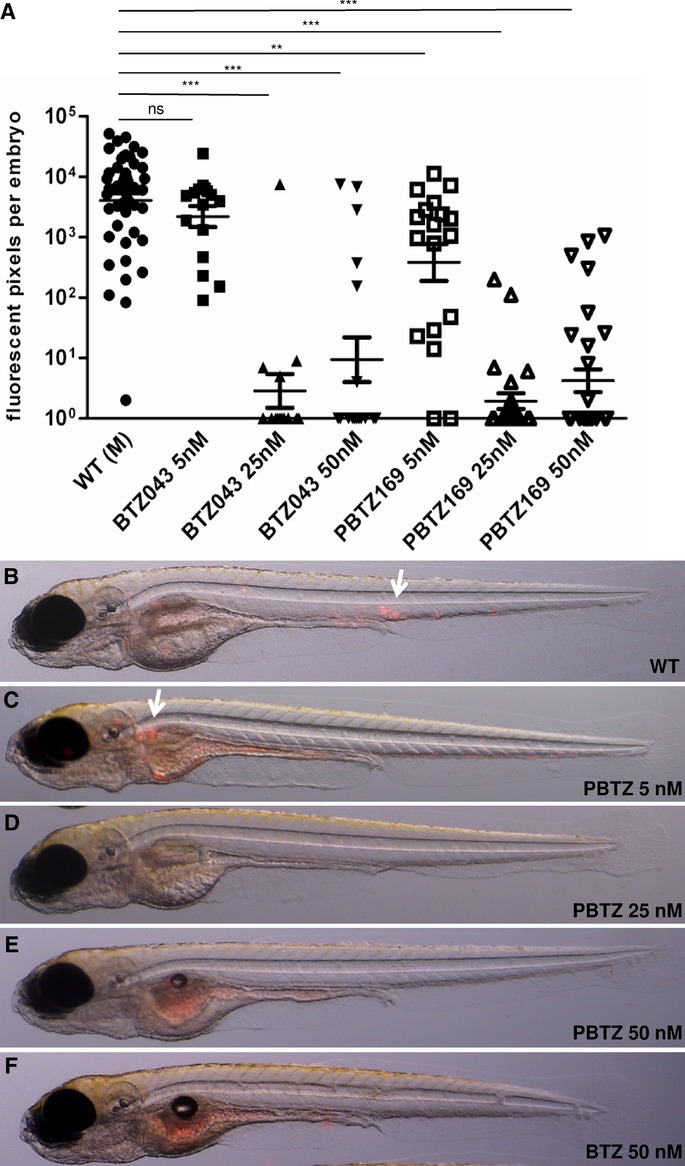
Treatment of M. marinum infected zebrafish embryos with BTZ. A One hour post infection (1 hpi) zebrafish embryos that had been infected with 50-200 CFU of fluorescent *M. marinum* were treated for 5 days with BTZ043 or PBTZ169 at the concentrations indicated. Bacterial burden was assessed by fluorescence microscopy using a Leica MZ16FA microscope and customized software to quantify infection levels. Graphs represent data from 2 - 8 independent experiments: ns, not significant; **P < 0.001; ***P < 0.0001; one-way ANOVA, Bonferroni's multiple comparison test. The M strain was used for infection. B–F Images of infected zebrafish embryos treated with PBTZ169 at 0 (WT), 5, 25 and 50 nM, or BTZ043 at 50 nM. The arrows indicate clusters of mycobacteria that were not present in fish treated with (P)BTZ at concentrations >25 nM. Note that the red fluorescence on the yolk/gut of the embryos is background staining.

To establish whether BTZ derivatives have the potential to cure other mycobacterial infections we tested their efficacy against *M. marinum* using the zebrafish embryo model as this has proved to be a powerful tool for assessing the effect of TB drugs (Davis *et al*, 2002; Adams *et al*, 2011). This model assesses simultaneously the effect of compounds on host survival, host pathology and bacterial burden. Embryos were infected with *M. marinum* strains E11 or M, producing the fluorescent protein mCherry, and observed by fluorescence microscopy. Treatment of infected zebrafish embryos with increasing concentrations of PBTZ169 or BTZ043 led to a decrease in the bacterial burden after 5 days, as measured by the amount of fluorescent pixels present in the embryos (Fig 4A). Whereas infection with *M. marinum* M (or E11 data not shown) resulted in substantial bacterial clustering (Fig 4B, C), almost no bacteria were present when infected zebrafish embryos were treated with 25 or 50 nM PBTZ169 or BTZ043 (Fig 4D, E, F). Treatment of embryos with either compound at 5 nM had no significant effect on the infection (Fig 4A, C).

**Figure 5:**
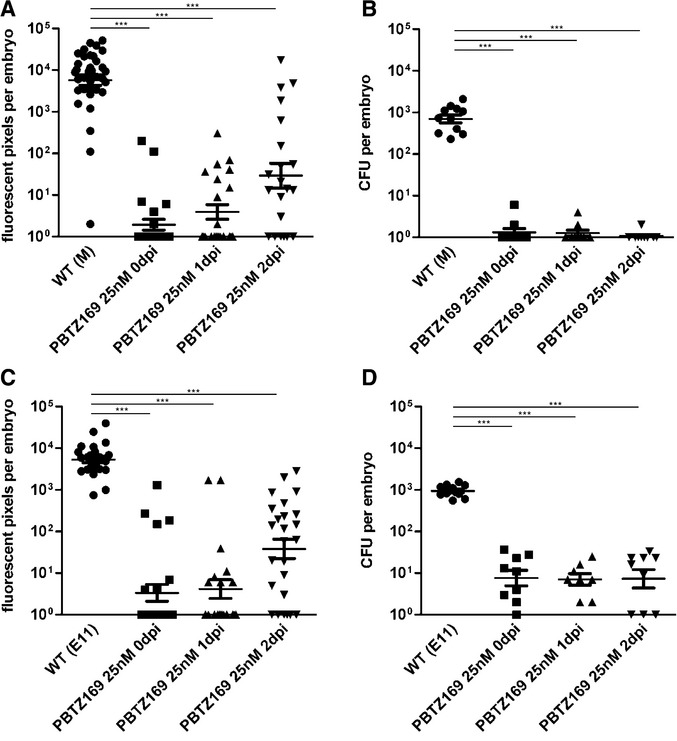
Effect of PBTZ169 treatment on infected zebrafish embryos. Zebrafish embryos were infected with either *M. marinum* strain M (A, B) or E11 (C, D). Treatment with PBTZ169 (25 μM) started at 0, 1 or 2 days post-infection (dpi) up to 5 dpi, giving exposure times of 5, 4 and 3 days, respectively. The bacterial burden was assessed by fluorescence microscopy and customized software to quantify the number of fluorescent pixels (A and C), and by enumeration of the CFU in lysed embryos (B and D), immediately after imaging was performed. In B and D, each data point represents the mean number of CFU per embryo of 3 embryo extracts plated, the bars indicate means and s.e.m. after log transformation. The graphs represent data from 2 to 4 independent experiments. ***P < 0.0001, one-way ANOVA, Bonferroni's multiple comparison test. A,B Zebrafish embryos infected with *M. marinum* strain M. C,D Zebrafish embryos infected with *M. marinum* strain E11.

To confirm the bactericidal effect, zebrafish embryos were infected with *M. marinum* strains M and E11, and then exposed to 25 nM PBTZ169 for 5, 4 or 3 days with the drug added at 0, 1 or 2 days post-infection, respectively. The number of colony forming units (CFU) per embryo was determined and compared to the level of fluorescence. A decrease of about 3 and 2 log units was observed in the number of CFU for the *M. marinum* strains M and E11, respectively (Fig 5B, D) independently of the duration of treatment. Decreasing bacterial viability was mirrored by a sharp decrease in fluorescence although more scatter was seen at later time-points (Fig 5A, C).

**Figure 6:**
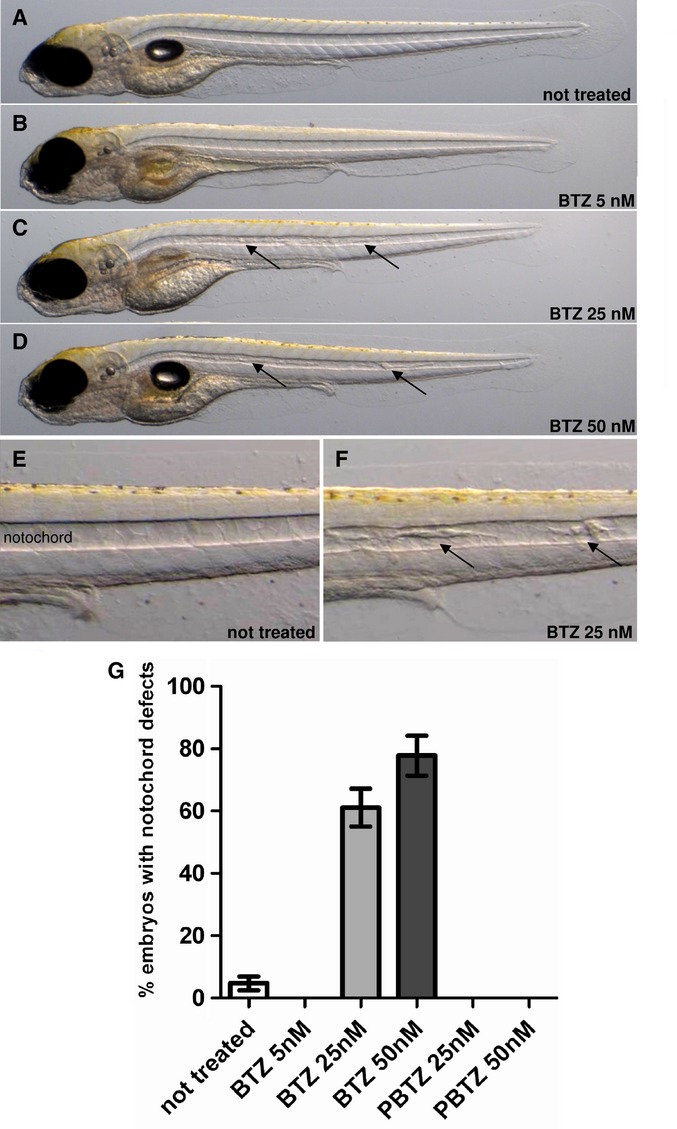
Effect of BTZ043 on M. marinum infected zebrafish embryo development. A–D *M. marinum* infected zebrafish embryos were treated with BTZ043 for 5 days at the concentrations indicated and photographed using a Leica DFC420C camera. The M strain was used for infection. E–F Images of the notochord from untreated and treated zebrafish, arrows indicate developmental abnormalities. G Analysis of zebrafish embryos displaying developmental abnormalities treated with BTZ043 (BTZ) or PBTZ169 (PBTZ) at the concentrations indicated.

On examination of infected zebrafish embryos treated with BTZ043 it was observed that the compound affected embryo development (Fig 6A–G) especially at concentrations above 25 nM. Administration of BTZ043 to the embryos 1 day after fertilization resulted in defects in notochord development and a slightly shortened Anterior-Posterior axis (Fig 6C, D) with 60.4% (*n* = 29 out of 48) of embryos affected after treatment with 25 nM BTZ043 and 76.7% (*n* = 23 out of 30) after treatment with 50 nM BTZ043 (Fig 6G). These defects were also observed when uninfected embryos were exposed to BTZ043. However, no developmental defects were seen after treatment with PBTZ169 at the same concentrations (compare Fig 4E, F) or even at 10 μM.

### Comparative efficacy of PBTZ derivatives *in vivo*

**Figure 7:**
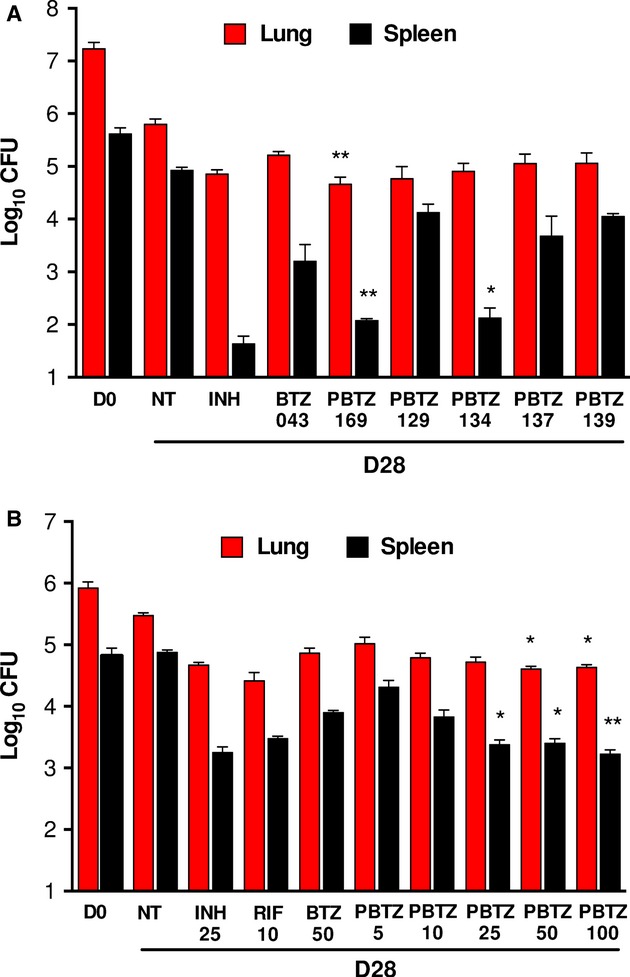
Efficacy studies in *vivo*.
Efficacy of 5 PBTZ candidates in a mouse model of chronic TB compared with INH (25 mg/kg), BTZ043 and untreated controls (NT). All BTZs were administered at 50 mg/kg of body weight per day. Red and black columns correspond to the bacterial burden in the lungs and spleens, respectively, at day 0 (D0) when treatment initiated, or day 28 (D28) when treatment finished. Bars represent the mean ± s.d. of CFUs from 5 mice per group. Significance in difference relative to BTZ043 were calculated using Student's *t*-test. *P <* *0.05; **P <* *0.005.Dose escalation study of PBTZ169 in the same model. 5 mice per group were treated with various drugs at the doses indicated (mg/kg). Colors, bars and statistics are as in (A).

The *in vivo* efficacy of PBTZ169 and four other candidates was assessed in the murine model of chronic TB after low-dose aerosol infection of BALB/c mice and treatment at 50 mg/kg, the recommended dose for BTZ043. Compared to the untreated control group, the bacterial burden in the lungs and spleens of BTZ043-treated mice was 0.6 and 1.7 logs lower, respectively (Fig 7A). All five PBTZs were active in both organs and not inferior to BTZ043. Strikingly, PBTZ169 and PBTZ134 reduced the bacterial burden in the spleens 10-fold more than BTZ043 did. Furthermore, PBTZ169 had significantly greater bactericidal activity in the lungs reducing the number of CFU by >0.5 log in comparison to BTZ043 at the same dose (Fig 7A). This activity was also equivalent to that of INH, suggesting that PBTZ169 was the most potent of all the BTZs *in vivo*.

### Dose escalation study and comparative pharmacokinetics

PBTZ169 was selected for further investigation and its efficacy *in vivo* compared to that of BTZ043 in a dose escalation study using the chronic model of TB. PBTZ169 was administered at 5, 10, 25, 50 and 100 mg/kg, whereas BTZ043 was given at 50 mg/kg. Four weeks of treatment with BTZ043 reduced the bacterial burden in the lungs and spleens by 1 log (Fig 7B). PBTZ169 was active at all the concentrations tested and had significantly greater bactericidal activity than BTZ043 at the same dose. Lowering the dose of PBTZ169 decreased the activity, but there was no significant difference in the bacterial burden in the lungs of mice treated with PBTZ169 at 5 mg/kg and BTZ043 at 50 mg/kg. Above 25 mg/kg, PBTZ169 was significantly better than BTZ043 at lowering the number of CFU in the spleen. PBTZ169 at 25 mg/kg displayed comparable bactericidal activity in both the lungs and spleens of mice (Fig 7B) to the frontline drug isoniazid (INH).

To investigate whether differential exposure was responsible for the differences observed between BTZ043 and PBTZ169 efficacy *in vivo*, compound pharmacokinetics were measured in mice orally dosed with 25 mg/kg of the respective compounds. The better efficacy of PBTZ169 cannot be accounted for by differences in the pharmacokinetics of the two compounds since, except for the faster uptake of PBTZ169 (Supplementary Fig 4), these behaved in a similar manner (Supplementary Table 5).

### Combination studies *in vitro* of PBTZ169 and other TB drugs

**Figure 8:**
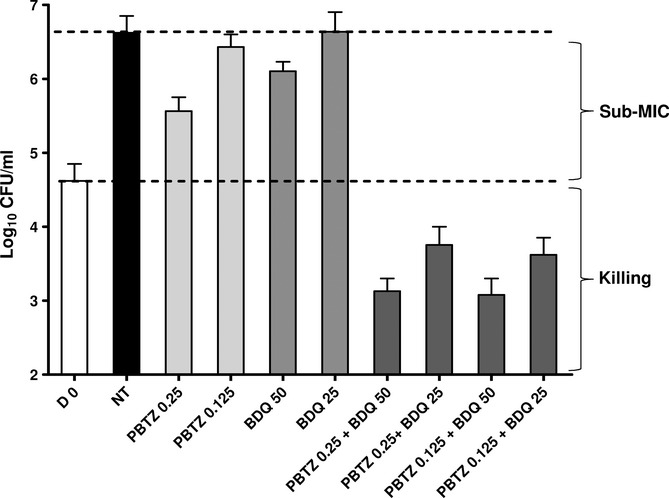
Synergistic interaction in vitro between BDQ and PBTZ169. The effect of serial dilutions of PBTZ169 and BDQ in combination on the viability of *M. tuberculosis* H37Rv was assessed after 7 days exposure. Bacterial dilutions were plated on solid medium and incubated for 1 month before counting the number of CFU. Drug concentrations are in ng/ml.

The REMA method was used to assess the viability of *M. tuberculosis* H37Rv after 7 days exposure to PBTZ169 alone and in combination with other approved and experimental TB drugs, as previously described (Lechartier *et al*, 2012). Additive effects were seen in combination with INH, MXF, PA-824, RIF and SQ109 but synergy was observed when PBTZ169 was combined with BDQ (Supplementary Fig 5). The combination of PBTZ169 and BDQ gave a ΣFIC (sum of the fractional inhibitory concentrations) of 0.5 using the checkerboard method indicating that the drugs act synergistically to inhibit growth of *M. tuberculosis* (Supplementary Fig 5). To confirm the bactericidal effect of this combination the number of CFU was determined after 7 days' exposure. The data show that the combination of 0.125 ng/ml of PBTZ169 and 25 ng/ml BDQ, which both have little or no impact on bacterial growth when used alone, show clear bactericidal activity when used in combination thereby confirming their synergistic interaction (Fig 8).

### Combination studies in the mouse model of chronic TB

**Figure 9:**
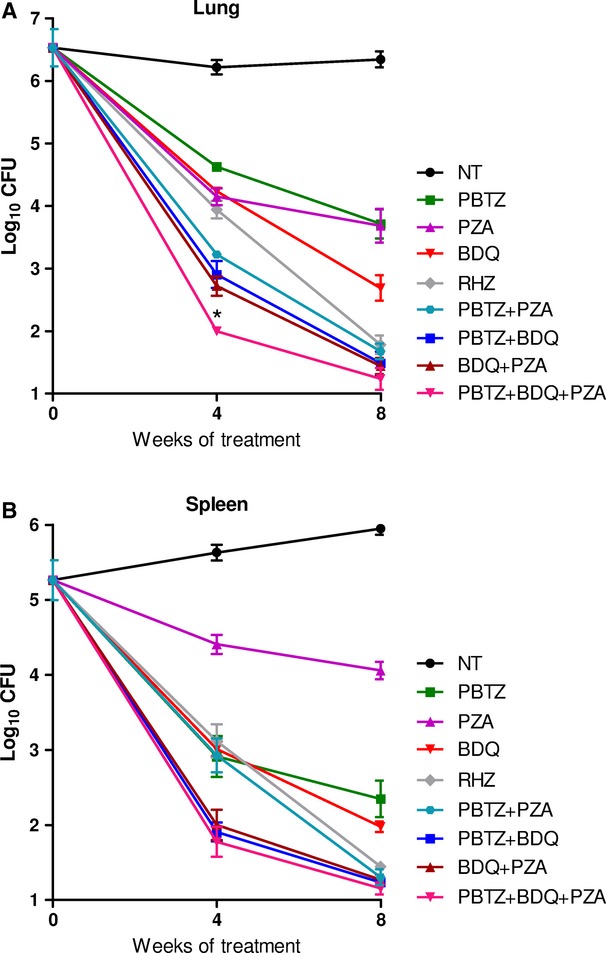
Efficacy of PBTZ169-based combination treatment in mice. The bactericidal effects of PBTZ, BDQ (both 25 mg/kg) and PZA (150 mg/kg) alone and in combination were compared in the lung (A) and in the spleen (B) with a standard regimen of RIF, INH and PZA (RHZ) and untreated controls (NT) in the murine model of chronic TB. The combination of PBTZ169 and BDQ plus PZA was significantly more effective than the standard treatment in reducing the number of CFU in both organs after 1 and 2 months of treatment (Supplementary Table 6). The lung CFU count of PBTZ-BDQ-PZA (*) was below the limit of detection used after 4 weeks of treatment (<200 CFU).

If PBTZ169 is to be used in a new regimen for TB treatment in humans it is important to demonstrate the efficacy of appropriate drug combinations in animal models. Consequently, we assessed the combination found to be synergistic *in vitro* in the murine model of chronic TB after low-dose aerosol infection. PBTZ169 was tested alone (at 25 mg/kg), in combination with BDQ (25 mg/kg) and pyrazinamide (PZA; 150 mg/kg), and with both drugs together, against *M. tuberculosis* H37Rv. The reduction in the bacterial burden in the lungs and spleens was measured after 4 and 8 weeks of treatment and compared to that obtained with the standard three drug therapy comprising INH, RIF and PZA at concentrations of 25, 10 and 150 mg/kg, respectively. As can be seen in Fig 9, the combination of PBTZ169 and BDQ was more effective than the standard treatment in reducing the number of CFU in both organs after 1 month of treatment (*P* values = 0.004 for the lung, 0.002 for the spleen) whereas the addition of PZA did not further improve the potency of the combination at this stage (*P *=* *0.003 for the spleen). The number of bacteria remaining in the lungs of mice treated with the experimental combination was below the limit of detection used at this time-point (<200 CFU). After 2 months of treatment (Fig 9), only the triple combination PBTZ, BDQ and PZA was significantly better than RHZ both in the lungs (*P *=* *0.046) and in the spleen (*P *=* *0.015; Supplementary Table 6). The efficacy of the combination of PBTZ, BDQ and PZA was thus superior to the standard triple therapy INH, RIF and PZA in the chronic model of TB.

## Discussion

Our goal was to use structure-activity-relationship (SAR) studies of BTZ to produce a preclinical candidate for subsequent use as a TB drug in humans. The addition of a piperazine to the BTZ scaffold resulted in even more potent derivatives, the PBTZ series that can also be formulated as salts. PBTZ169 proved to be the most suitable candidate for development and this was intensively investigated alongside the first generation lead, BTZ043. While the mechanism of action of both compounds was identical, inhibition of DprE1 function by formation of a covalent bond to the active site cysteine residue, PBTZ169 has many superior features.

First, there are no chiral centers in the compound so the synthesis, quality control and, above all, manufacturing will be less expensive, an important factor for a disease that mainly concerns the developing world. Second, PBTZ169 is less cytotoxic than BTZ043 and showed significantly better efficacy at lower concentrations in the murine model of chronic TB (Fig 7) and this may be attributed to the fact that PBTZ169 inhibits DprE1 more efficiently than BTZ043 does. There was a notable reduction in bacterial loads in the lungs and, especially, the spleen following treatment with PBTZ169 and PBTZ134, compared to BTZ043 treatment, and these two drug candidates are as potent as INH. Third, the better protection of PBTZ169 from nitroreductases (Fig 2) may eventually be important as other microorganisms (e.g. in the human digestive tract) or host enzymes could also reduce benzothiazinones to their inactive hydroxylamino or amino forms.

Differences in pharmacokinetics cannot account for the superior behavior of PBTZ169 (Supplementary Fig 4) as both compounds displayed similar properties. Pharmacokinetic studies showed that PBTZ169 appears to be absorbed more rapidly than BTZ043 with absorption likely occurring in the stomach as well as in the intestines. The better solubility of PBTZ169 in acidic conditions may account for this. Furthermore, from the exposure profile of PBTZ169 in mice it appears that once daily dosing should be sufficient in humans as the serum level remains well above MIC for >21 h.

From an SAR standpoint, the availability of co-crystal structures of DprE1 with both BTZ043 (Neres *et al*, 2012) and PBTZ169 also helps our understanding. The *M. tuberculosis* DprE1 structure with PBTZ169 shows that binding of this benzothiazinone is in full agreement with the previously reported *M. smegmatis* DprE1 in complex with BTZ043 (Neres *et al*, 2012). The benzothiazinone rings of BTZ043 and PBTZ169 adopt identical positions in the active site of DprE1. The greater flexibility of the cyclohexylmethyl-piperazine moiety of PBTZ169, making it more adaptable to the active site when compared to BTZ043, might account in part for its higher affinity and faster inactivation of DprE1. In addition, the likely protonation of one of the piperazine ring nitrogen atoms of PBTZ169 could generate a favourable interaction with two negatively charged residues, namely Asp318 and Glu322, present in the disordered loop (residues 314-328) located over the active site. Information obtained here about the localization of FPX in the DprE1 active site will also facilitate understanding of the enzymatic reaction and aid rational drug design.

Another potentially important difference between PBTZ169 and BTZ043 was detected through use of the zebrafish embryo model following infection with *M. marinum*. Although both compounds lowered the bacterial burden in a dose-dependent manner, exposure to BTZ043 seemingly led to developmental abnormalities (Fig 6). These included the appearance of deposits in the notochord and the subsequent shortening of the Anterior-Posterior axis resulting in shorter body length although the reason for this is unknown. When PBTZ169 was tested in this model no such developmental defects were observed making this the preferred compound for clinical development.

Of considerable significance with respect to the ultimate objective of curing disease in humans was the full compatibility of PBTZ169, and BTZ043 (Lechartier *et al*, 2012), with all the other approved and experimental TB drugs tested. The synergy between PBTZ169 and BDQ was particularly noteworthy and may be explained in two possible ways; weakening of the cell wall by DprE1 inhibition leading to better penetration of BDQ and easier access to its target, ATP synthase. Alternatively, loss of DprE1 activity, due to binding of PBTZ169, might result in fewer reducing equivalents entering the electron transfer chain from FADH_2_.

Full compatibility with other drug candidates means that novel combinations including PBTZ169 can be readily assembled and this is consistent with the current thinking that it may be more efficient and less time-consuming to test new regimens in clinical trials rather than testing a series of single drugs separately (Diacon *et al*, 2012). Highly encouraging results were obtained against chronic murine TB when PBTZ169 was combined with BDQ or PZA or both drugs (Fig 9) as these combinations reduced the bacterial load more rapidly than the current tri-therapy (RIF, INH, PZA). It is now urgent to complete the preclinical development of PBTZ169, since it is an attractive, well-understood drug candidate that offers great potential not only for the control of TB but also for other mycobacterial diseases, such as leprosy and Buruli ulcer, as well as for related infections like Nocardiosis (Makarov *et al*, 2006; Vera-Cabrera *et al*, 2012).

## Materials and Methods

### Chemistry

Standard procedures were applied and the synthetic route for PBTZ compounds is described in the supporting information (Makarov *et al*, 2006; Makarov, 2011). All compounds were at least 96% pure.

#### Bacterial strains and culture conditions

Bacterial strains, BTZ-resistant mycobacterial mutants and *M. tuberculosis* strain H37Rv were grown at 37°C with shaking in Middlebrook 7H9 (Difco) broth supplemented with 10% albumin-dextrose-catalase (ADC) enrichment, 0.2% glycerol, 0.05% Tween 80. The *M. marinum* strains E11 (Puttinaowarat *et al*, 2000) and M ((Stinear *et al*, 2008)ATCC BAA-535) containing the plasmid pSMT3-mcherry (Meijer *et al*, 2008) to visualize bacteria, were routinely grown at 30°C in Middlebrook 7H9 broth (Difco) with 10% Middlebrook albumin-dextrose-catalase (ADC, BD Bioscience) and 0.05% Tween-80 by shaking at 90 rpm or on Middlebrook 7H10 agar (Difco) supplemented with 10% oleic acid-albumin-dextrose-catalase (OADC, BD Bioscience) and 50 mg/ml hygromycin.

#### Antimicrobials

Drugs were purchased from Sigma or provided by Tibotec (BDQ). PBTZ derivatives were suspended in 0.5% carboxymethyl cellulose for the comparative efficacy studies. PBTZ169 and BDQ were suspended in 20% hydroxypropyl-β-cyclodextrin (pH = 3.0) for *in vivo* combination studies. Solutions of compounds for administration to mice were prepared weekly and stored at 4°C for all compounds but BDQ which was prepared monthly and stored at 4°C. PZA, INH, and RIF were suspended in water.

### Biochemistry and structural biology

NfnB assays were performed at 25°C, as outlined previously (Manina *et al*, 2010). Briefly, compounds were added to a reaction mixture containing NfnB (6 μM), NADH (150 μM), 50 mM Tris-HCl pH 8.0, 150 mM NaCl, and 5% glycerol. Full details of the purification and crystallization of *M. tuberculosis* DprE1 are given in the supporting information. DprE1 inhibition was assessed following incubation with BTZ043 or PBTZ169 (0–20 μM) for 5 min, using a peroxidase-coupled assay with Amplex Red as a substrate. The enzyme (5 μM) was incubated at 30°C with inhibitor and 200 μM FPR, in 50 mM glycylglycine pH 8.4, 100 mM NaCl. An aliquot (5 μL) was taken after 5 min incubation and diluted assay mixture (final volume 50 μL) to give final concentrations of 400 μM FPR, 0.2 μM horseradish peroxidase and 50 μM Amplex Red and 0.5 μM DprE1. The peroxidase activity was then assessed by continuous measurement of the fluorescence with excitation/emission wavelengths of 560/590 nm, respectively. Analysis of DprE1-PBTZ169 complexes by mass spectrometry was performed as reported previously, now using *M. tuberculosis* DprE1 (Neres *et al*, 2012).

### Zebrafish infection

The zebrafish (*Danio rerio*) embryo infection experiments were performed largely as described previously (Stoop *et al*, 2011). Volumes of 1 nl of bacterial suspension, containing 50-200 CFU were injected into the caudal vein of embryonic zebrafish at approximately 28 h post fertilization. At 5 days post infection, embryos were monitored using fluorescence microscopy (Leica MZ16FA). Brightfield and fluorescent images were generated with a Leica DFC420C camera and subsequently fluorescent images were analyzed with customized software to quantify infection levels. An updated version of the previously described software was used (see http://bio-imaging.liacs.nl/galleries/granulomaload/).

### Zebrafish drug treatment

Twelve infected embryos were incubated at 28°C in petri dishes of 4 cm diameter containing 4 ml sterilized egg water (60 μg/ml instant ocean sea salts) with 0.003% 1-phenyl-2-thiourea (Sigma) to prevent melanization. All drug treatment experiments were performed in egg water containing 1% DMSO and analyzed at 5 days post infection (dpi). Compound concentrations were tested at least three times. Compounds were administered once to the egg water either at 1 h post infection (hpi), 1 day post infection (dpi) or 2 dpi. The embryos remained in the same 4 ml egg water for the duration of the experiments, no daily refreshments of the egg water nor additions of more compound were performed. All procedures involving zebrafish embryos were executed in compliance with local animal welfare laws.

### Determination of bacterial loads of infected zebrafish embryos

Embryo lysates were prepared as described previously (Stoop *et al*, 2011). Briefly, pools of three embryos were dissociated in the presence of 5% (w/v) SDS, lysed and decontaminated by incubation with MycoPrep reagent (BD Bioscience), neutralized with phosphate buffered saline (PBS) then serial dilutions were plated.

### Murine infection models, treatment and assessment of efficacy

Female BALB/c mice, aged 5–6 weeks, were obtained from Charles River Laboratories. The *in vivo* efficacy of single drugs and combinations was assessed 4 weeks after a low-dose aerosol infection of mice in the chronic model of TB, by gavage 5 days a week for 4 weeks. Experiments were approved by the Swiss Cantonal Veterinary Authority (authorization no. 2218).

Drug treatment began 4 weeks after infection at the following doses (mg/kg): BDQ, 25; PBTZ, 25; PZA, 150; RIF, 10; INH, 25. Control and treated mice were sacrificed, the lungs and spleens homogenized, and dilutions plated on 7H10 agar enriched with 10% OADC and supplemented with cycloheximide (10 μg/ml), ampicillin (85 μg/ml) and 0.4% w/v of activated charcoal (Sigma) to prevent compound carry-over. The plates were incubated for 35 days at 37°C before CFU were enumerated. CFU counts were log_10_ transformed before analysis as mean log_10_ CFU ± s.d., and compared using Student's t tests in Prism version 5.0 (Graphpad).

**The paper explained**

**Problem**

Tuberculosis is a continuing source of human morbidity and mortality, claiming over 1.4 million lives in 2012. The drugs currently used as part of combination therapy to treat the disease are old and somewhat inefficient by today's standards. Furthermore, treatment is compounded by widespread resistance to both frontline and second-line drugs due to infection with multidrug- and extensively drug-resistant strains of *Mycobacterium tuberculosis*, respectively. As a direct consequence of these failings there is a great need to discover and to develop new drugs and drug combinations.

**Results**

Here, we disclose the next generation of benzothiazinone compounds and present an optimized preclinical candidate, PBTZ169, that is highly potent against drug-susceptible and drug-resistant *Mycobacterium tuberculosis*. Like other benzothiazinones, PBTZ169 acts by forming a covalent bond to an active site cysteine residue in an enzyme essential for the production of the mycobacterial cell wall. The three-dimensional structure of PBTZ169 in complex with its target has been solved and used to explain the action of this drug candidate. More importantly, PBTZ169 was shown to be most effective at treating tuberculosis in two different animal models and to be compatible with both existing and experimental tuberculosis drugs.

**Impact**

In this work we present an advanced preclinical candidate drug for the treatment of tuberculosis in humans. PBTZ169 has been extensively characterized in terms of its chemical, biochemical, microbiological and pharmacokinetic properties. Of capital importance is the synergistic behaviour of PBTZ169 and bedaquiline, a drug recently approved for the treatment of multidrug-resistant tuberculosis, and with the classical drug pyrazinamide. The available data suggest that a combination of these three molecules should be highly efficacious in treating all forms of tuberculosis in humans.
