# Supplementary material for: Towards a new combination therapy for tuberculosis with next generation benzothiazinones
Source: EMBO Mol Med. 2014 Feb 5;6(3):372–83. doi: 10.1002/emmm.201303575 (PMC3958311; doi:10.1002/emmm.201303575)
Supplement: Supplementary file 1 [file emmm0006-0372-sd1.pdf]

Towards a new combination therapy for tuberculosis with next generation benzothiazinones

Vadim Makarov, Benoit Lechartier, Ming Zhang, João Neres, Astrid M. van der Sar, Susanne A. Raadsen, Ruben C. Hartkoorn, Olga B. Ryabova, Anthony Vocat, Laurent A. Decosterd, Nicolas Widmer, Thierry Buclin, Wilbert Bitter, Koen Andries, Florence Pojer, Paul J. Dyson & Stewart T. Cole

Corresponding author: Stewart Cole, Global Health Institute

Review timeline:

Submission date:	15 October 2013
Editorial Decision:	15 November 2013
Revision received:	04 December 103
Accepted:	10 December 2013

Transaction Report:

(Note: With the exception of the correction of typographical or spelling errors that could be a source of ambiguity, letters and reports are not edited. The original formatting of letters and referee reports may not be reflected in this compilation.)

Editor: Céline Carret

1st Editorial Decision 15 November 2013

Thank you for the submission of your manuscript to EMBO Molecular Medicine. We have now heard back from the three referees whom we asked to evaluate your manuscript.

As you will see from the below reports, the three referees are supportive of publication. Nevertheless, while referee 2 is only asking for fuller documentation disclosure, referee 3 would like to see the PBTZ0169 tested against multi-drug resistant strains of TB, better explanation of the zebrafish experiment, including evaluation of the drug concentration remaining in infected fish after treatment and a discussion regarding why atypical *Mycobacteria* seem insensitive to the drug.

Given these evaluations, I would be happy to consider a revised version of your manuscript, with the understanding that the referee concerns must be fully addressed and that acceptance of the manuscript may entail a second round of review.

Please see below for further editorial additions.

I look forward to seeing a revised form of your manuscript as soon as possible.

***** Reviewer's comments *****

Referee #1 (Remarks):

The paper by Makarov et al. reports on a new series of benzothiazinones and characterizes these compounds at a biochemical, structural and in appropriate models of TB infection. Potentially the authors have uncovered a TB regimen (PBTZ169-BDQ-PZA) that warrants further investigation in humans. The PBTZ169 compound is a major advance over BTZ043 and its synergy with BDQ is a major finding for developing drug combinations. The manuscript is a complete story and a significant contribution to the area of TB drug discovery. The paper is extremely well-written, easy to follow and error-free.

Referee #2 (Comments on Novelty/Model System):

The study includes both zebrafish and murine models to demonstrate in vivo efficacy. There are no concerns and the studies have been performed with rigor.

Referee #2 (Remarks):

The paper by Makarov and colleagues details analysis of new analogues of the benzothiazinones that inhibit the Mycobacterium tuberculosis enzyme decaprenylphosphoryl-ribose epimerase DprE1. DprE1 is important for the synthesis of arabinose which is essential for the manufacture of the abundant cell wall components lipoarabinomannan and arabinogalactan.
The inhibitors are based on previously identified structures and the target, DprE1, has been identified by several laboratories as an essential enzyme in Mtb. The novelty in the current study lies in the improved performance of the analogues and their interrogation in different biological readouts and in combination with various drug regimens. The information contained in this paper is of considerable value to the field and worthy of publication. My comments are extremely minor and focused more on improving the paper rather than correcting any perceived defects.

1. The scoring of mCherry fluorescence in the infected zebrafish is fraught with many challenges due to the dispersed nature of the signal, the "depth" of the fish, and differential quenching by the different tissues. For that reason the cfu data are absolutely critical and should be included in the figure and not shunted into supplementary data.
2. The PBTTZ169/BDQ synergy data from the REMA analysis should be added to the supplementary data to fully document the argument for synergy of action because this is very important in the design of combinatorial therapies moving forward. Personally, I'd like all the drug/drug data alluded to in this paragraph to be accessible in supplementary material.

Referee #3 (Comments on Novelty/Model System):

The manuscript by Makarov et al. describes the synthesis of a new series of benzothiazinone analogues, and compares the biochemical/pharmacological characterisitics of one of them, PBTZ169 with those of the parental BTZ043 compound. The study includes a wide array of techniques allowing the authors to propose PBTZ169 as an attractive preclinical candidate against TB.
Resolving the crystal structure of the DprE1-PBTZ169 complex revealed an identical mechanism of action between BTZ043 and PBTZ169, both targetting the decaprenylphosphoryl-ribose-epimerase. The in vivo efficacy of PBTZ1369 was then demontrated in two different animal models of tuberculosis: the zebrafish infected with M. marinum and the mouse model of chronic disease. Importantly, pharmacological studies indicated that PBTZ169 offeres improved safety and efficacy

compared to BTZ043. In addition, this compound showed additive or synergistic activity when combined with other TB drugs. A new regimen comprising PBTZ169, BDQ and PZA was found to be more efficacious than the standard three drug treatment (INH+ RIF+PZA).
This is an important work that addresses an important topic. However, I feel that several points need to be clarified to support the conclusions raised prior to publication.

Referee #3 (Remarks):

This is a well written manuscript dealing with an important topic. The experiments are well performed with all appropriate controls. However, I have a few substantial concerns that need to be addressed to support the main conclusions raised.

1. The authors propose PBTZ0169 as a new clinical candidate. As an attractive and well-understood drug candidate offering great potential to control TB, it remains essential to test its activity against multiple MDR and XDR strains. In their introduction, the authors have emphasized the increase number of MDR/XDR TB cases that are resistant to key front-line and second-line drugs. Table 2 shows the activity of PBTZ169 against numerous mycobacterial species, but surpisingly PBTZ169 was only tested against H37Rv as well as the NTB1 strain that contains a missense mutation C387S in DprE1. Therefore, activities against a panel of MDR/XDR strains need to be determined.

2. The Material and Methods does not describe in details how the treatment of infected ZF was done. Were the embryos treated for 5 days by changing the drug at a daily basis ? The legend of figure 4 should be better described.
What is the effective drug concentration within the infected ZF ? To estimate the efficacy of PBTZ169 in this model, it is important to evaluate the drug concentration present in the infected fish after 5 days of treatment. This could be easily performed using the LC-MS method developed by the authors to detect PBTZ169.

3. No explanation is given to explain why some atypical mycobacteria, in particular, M. abscessus and M avium, are fully resistant to PBTZ169. Did the authors align/compare the DprE1 sequence of M. tuberculosis atypical mycboacteria ? Could sequence differences (perhaps absence of Cys387 ?) explain the unability of PBTZ169 to form an irreversible adduct with DprE1 in these species ? Alternatively, is it due, like in M. smegmatis, to the presence of an NfnB-like nitroreductase that reduces the drug?

Minor points:
-The authors successfully used the zebrafish model of infection to evaluate the in vivo activity of their drug compounds. However, they have totally omitted to refer to the major contribution of the Ramakrishnan's lab where this model was established. Several recent papers have described the model to test the antitubercular activity of molecules in ZF embryos. These references should be added and discussed in the light of their own results (Adams et al. 2011. Cell 145:39-53; Takaki et al 2012. Cell Rep 2:175-84; Takaki et al 2013, Nature Protoc. 8:1114-24).

-page 8, line 267. The only reference about the ZF model is not correct. The Swaim et al 2006 mainly deals with adult ZF. It should be more appropriate to refer to the original paper by Davis et al. 2002, Immunity 17:693-702.

1st Revision - authors' response 04 December 103

***** Reviewer's comments *****

Referee #1 (Remarks):

The paper by Makarov et al. reports on a new series of benzothiazinones and characterizes these compounds at a biochemical, structural and in appropriate models of TB infection. Potentially the

authors have uncovered a TB regimen (PBTZ169-BDQ-PZA) that warrants further investigation in humans. The PBTZ169 compound is a major advance over BTZ043 and its synergy with BDQ is a major finding for developing drug combinations. The manuscript is a complete story and a significant contribution to the area of TB drug discovery. The paper is extremely well-written, easy to follow and error-free.

Response

We thank Referee #1 for his/her evaluation and appreciation of our work.

Referee #2 (Comments on Novelty/Model System):

The study includes both zebra fish and murine models to demonstrate in vivo efficacy. There are no concerns and the studies have been performed with rigor.

Referee #2 (Remarks):

The paper by Makarov and colleagues details analysis of new analogues of the benzothiazinones that inhibit the Mycobacterium tuberculosis enzyme decaprenylphosphoryl-ribose epimerase DprE1. DprE1 is important for the synthesis of arabinose which is essential for the manufacture of the abundant cell wall components lipoarabinomannan and arabinogalactan.

The inhibitors are based on previously identified structures and the target, DprE1, has been identified by several laboratories as an essential enzyme in Mtb. The novelty in the current study lies in the improved performance of the analogues and their interrogation in different biological readouts and in combination with various drug regimens. The information contained in this paper is of considerable value to the field and worthy of publication. My comments are extremely minor and focused more on improving the paper rather than correcting any perceived defects.

1. The scoring of mCherry fluorescence in the infected zebra fish is fraught with many challenges due to the dispersed nature of the signal, the "depth" of the fish, and differential quenching by the different tissues. For that reason the cfu data are absolutely critical and should be included in the figure and not shunted into supplementary data.

Response

We have followed this suggestion by including the CFU data and moving the figure from the supplementary material into the main text. There are now 9 figures instead of 8.

2. The PBTTZ169/BDQ synergy data from the REMA analysis should be added to the supplementary data to fully document the argument for synergy of action because this is very important in the design of combinatorial therapies moving forward. Personally, I'd like all the drug/drug data alluded to in this paragraph to be accessible in supplementary material.

Response

We have included an additional figure in the supplementary material that shows the primary data from REMA analysis as a figure. All of the PBTZ169/BDQ data are now either in the main text or the supplementary material.

Referee #3 (Comments on Novelty/Model System):

The manuscript by Makarov et al. describes the synthesis of a new series of benzothiazinone analogues, and compares the biochemical/pharmacological characteristics of one of them, PBTZ169 with those of the parental BTZ043 compound. The study includes a wide array of techniques allowing the authors to propose PBTZ169 as an attractive preclinical candidate against TB.

Resolving the crystal structure of the DprE1-PBTZ169 complex revealed an identical mechanism of action between BTZ043 and PBTZ169, both targeting the decaprenylphosphoryl-ribose-epimerase. The in vivo efficacy of PBTZ1369 was then demonstrated in two different animal models of tuberculosis: the zebra fish infected with M. marinum and the mouse model of chronic disease. Importantly, pharmacological studies indicated that PBTZ169 offers improved safety and efficacy compared to BTZ043. In addition, this compound showed additive or synergistic activity when combined with other TB drugs. A new regimen comprising PBTZ169, BDQ and PZA was found to be more efficacious than the standard three drug treatment (INH+ RIF+PZA).

This is an important work that addresses an important topic. However, I feel that several points need to be clarified to support the conclusions raised prior to publication.

Referee #3 (Remarks):

This is a well-written manuscript dealing with an important topic. The experiments are well performed with all appropriate controls. However, I have a few substantial concerns that need to be addressed to support the main conclusions raised.

1. The authors propose PBTZ0169 as a new clinical candidate. As an attractive and well-understood drug candidate offering great potential to control TB, it remains essential to test its activity against multiple MDR and XDR strains. In their introduction, the authors have emphasized the increase number of MDR/XDR TB cases that are resistant to key front-line and second-line drugs. Table 2 shows the activity of PBTZ169 against numerous mycobacterial species, but surprisingly PBTZ169 was only tested against H37Rv as well as the NTB1 strain that contains a missense mutation C387S in DprE1. Therefore, activities against a panel of MDR/XDR strains need to be determined.

Response

We thank the referee for pointing out this oversight. In previous work, we tested over 200 clinical isolates of *M. tuberculosis* for their susceptibility to BTZ043 and other BTZ derivatives. This included a range of drug-susceptible and MDR- and XDR-isolates all of which were found to be BTZ-susceptible. Susceptibility is class-specific and not compound-specific although there are obviously differences in activity between different BTZ derivatives.

We have now included the findings with PBTZ169 in the form of an additional row in Table 2 (highlighted in yellow) and a new supplementary Table (Table S2). Furthermore, a short statement describing the PBTZ169-susceptibility of drug-resistant clinical isolates has now been included on lines 176-178:

"Consistent with previous findings (Makarov et al, 2009; Pasca et al, 2010), both PBTZ169 and BTZ043 were active against MDR- and XDR-clinical isolates of *M. tuberculosis* (Table 2; Table S2)."

2. The Material and Methods does not describe in details how the treatment of infected ZF was done. Were the embryos treated for 5 days by changing the drug at a daily basis ? The legend of figure 4 should be better described.

Response

We apologize for the lack of experimental detail and have now rewritten the corresponding section

in the Material and Methods section viz:

"Compounds were administered once to the egg water either at 1 hour post infection (hpi), 1 day post infection (dpi) or 2 dpi. The embryos remained in the same 4 ml egg water for the duration of the experiments, no daily refreshments of the egg water nor additions of more compound were performed. All procedures involving zebra fish embryos were executed in compliance with local animal welfare laws."

Furthermore, we have expanded the legend to Figure 4 in the interest of clarity.

What is the effective drug concentration within the infected ZF ? To estimate the efficacy of PBTZ169 in this model, it is important to evaluate the drug concentration present in the infected fish after 5 days of treatment. This could be easily performed using the LC-MS method developed by the authors to detect PBTZ169.

Response

The referee is right to suggest that it would be interesting to know the drug concentrations in infected zebra fish to assess the efficacy of PBTZ169 in this model. However, these pharmacokinetic (PK) experiments are not easily performed; we have estimated that, with the currently available techniques, we need over 300 zebra fish embryos for a single measurement per treatment per time-point. We also would like to point out that in none of the high-impact papers on zebra fish antibiotic screening that this referee suggests we cite (see below) have such measurements been performed, which indicates that these experiments are (i) probably more difficult than might seem and (ii) not standard in these kind of studies.

In the event that no efficacy of PBTZ169 had been seen against *M. marinum* in the zebra fish embryo we might have undertaken a pharmacokinetic analysis in order to find an explanation. As it was, both BTZ043 and PBTZ169 both showed very good efficacy in dose ranging studies in this disease model thus removing this requirement and demonstrating that BTZ derivatives are active in a second animal model after mice.

It was clearly more important to conduct PK analysis in mice in order to establish the optimal dose and to be able to perform combination studies of PBTZ169 with other drugs in the generally accepted animal model for chronic TB. This important information is included both in the paper and the Supporting Information.

3. No explanation is given to explain why some atypical mycobacteria, in particular, M. abscessus and M avium, are fully resistant to PBTZ169. Did the authors align/compare the DprE1 sequence of M. tuberculosis atypical mycboacteria ? Could sequence differences (perhaps absence of Cys387 ?) explain the inability of PBTZ169 to form an irreversible adduct with DprE1 in these species ? Alternatively, is it due, like in M. smegmatis, to the presence of an NfnB-like nitroreductase that reduces the drug?

Response

The referee's interpretation of the natural resistance of certain mycobacteria to BTZ compounds is correct. These species have *dprE1* genes where the codon equivalent to Cys387 of *M. tuberculosis* has been replaced by an Ala or Ser codon. A statement to this effect has now been added to the text in lines 172-176:

"PBTZ169 and BTZ043 were not active against atypical mycobacteria like *M. avium* (Makarov et al, 2009) nor against *M. abscessus, M. boletti*, *M. massiliense* and *M. vaccae* (Table 2) probably because these mycobacteria possess *dprE1* genes where the codon equivalent to Cys387 of *M. tuberculosis* has been replaced by an Ala or Ser codon (see below)."

Minor points:

-The authors successfully used the zebra fish model of infection to evaluate the in vivo activity of their drug compounds. However, they have totally omitted to refer to the major contribution of the

Ramakrishnan's lab where this model was established. Several recent papers have described the model to test the antitubercular activity of molecules in ZF embryos. These references should be added and discussed in the light of their own results (Adams et al. 2011. Cell 145:39-53; Takaki et al 2012. Cell Rep 2:175-84; Takaki et al 2013, Nature Protoc. 8:1114-24).

Response

As the zebra fish model played a relatively modest role in our paper we do not consider it necessary to discuss this at any great length nor to engage in extensive comparisons. However, we have acknowledged the contributions of the Ramakrishnan laboratory in this respect by citing and briefly discussing the Adams paper and have also replaced the Swaim paper (from the Ramakrishnan lab) on the former line 267 with the Davis et al. 2002 reference viz:

"To establish whether BTZ derivatives have the potential to cure other mycobacterial infections we tested their efficacy against *M. marinum* using the zebra fish embryo model as this has proved to be a powerful tool for assessing the effect of TB drugs (Adams et al, 2011; Davis et al, 2002)."

-page 8, line 267. The only reference about the ZF model is not correct. The Swaim et al 2006 mainly deals with adult ZF. It should be more appropriate to refer to the original paper by Davis et al. 2002, Immunity 17:693-702.

Response

See point immediately above. We thank the referee for proposing this.

© EMBO
