# Supplementary material for: Towards a new combination therapy for tuberculosis with next generation benzothiazinones
Source: EMBO Mol Med. 2014 Feb 5;6(3):372–83. doi: 10.1002/emmm.201303575 (PMC3958311; doi:10.1002/emmm.201303575)
Supplement: Supplementary file 2 [file emmm0006-0372-sd2.pdf]

Supplementary figures

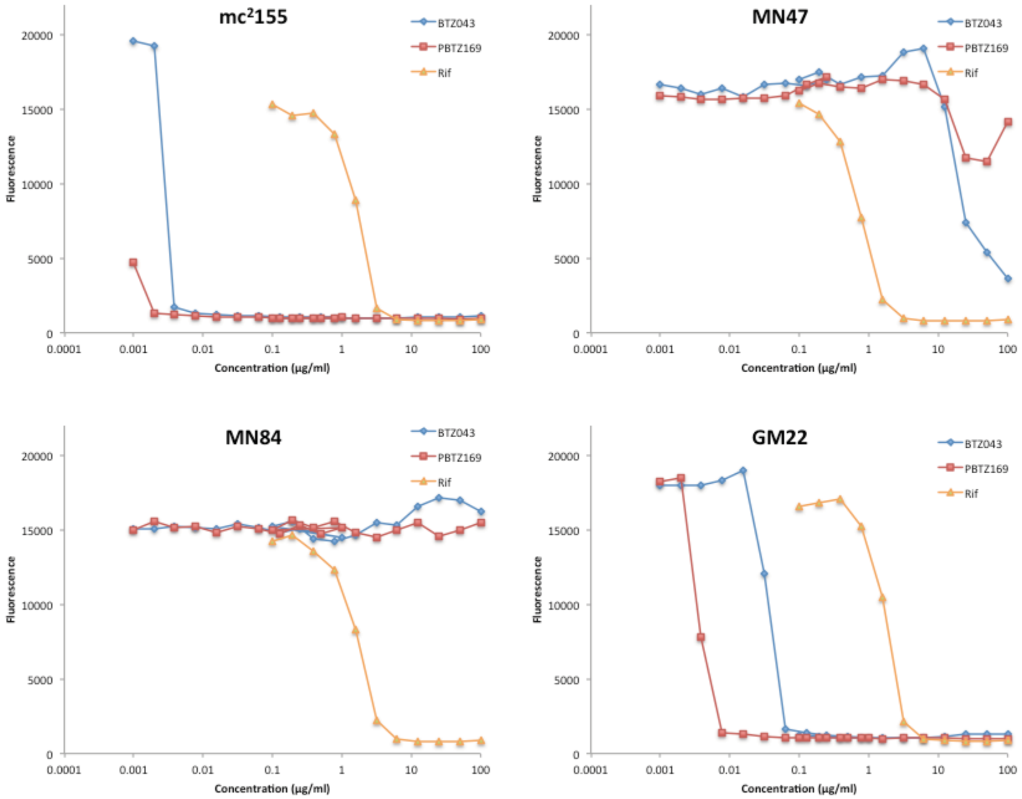

Figure S1. Susceptibility of BTZ-resistant mutants of *M. smegmatis* to PBTZ169. Susceptibility was measured using REMA with fluorescence units shown on the Y-axis. **A.** Wild-type parental strain mc^{2}155; **B.** BTZ-resistant mutant MN47 (Cys394Ser); **C.** BTZ-resistant mutant with MN84 (Cys394Ser); **D.** BTZ-resistant mutant GM22 overproducing NfnB nitroreductase.
