# Supplementary material for: Towards a new combination therapy for tuberculosis with next generation benzothiazinones
Source: EMBO Mol Med. 2014 Feb 5;6(3):372–83. doi: 10.1002/emmm.201303575 (PMC3958311; doi:10.1002/emmm.201303575)
Supplement: Supplementary file 3 [file emmm0006-0372-sd3.pdf]

A.

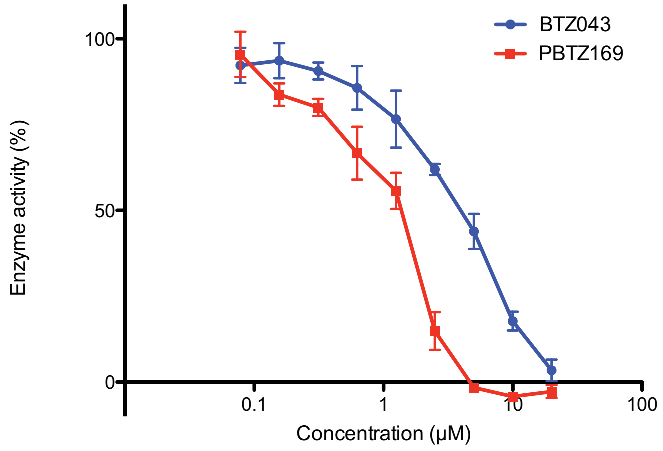

B.

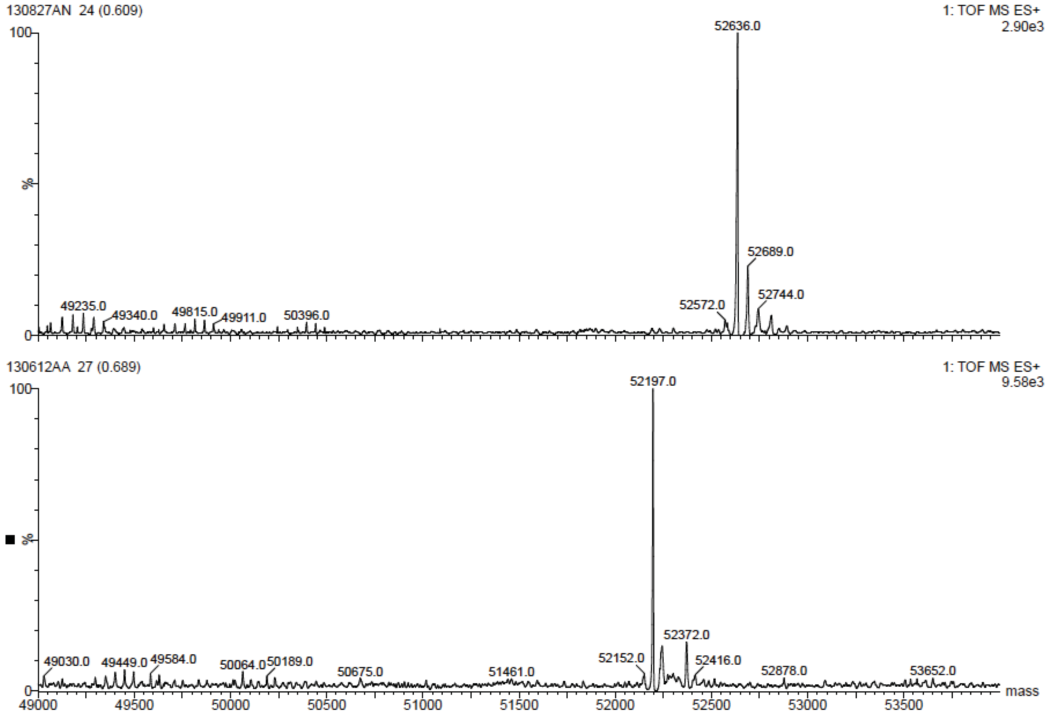

Figure S2. Enzyme inhibition and mass spectrometry analysis of the covalent DprE1-inhibitor adducts. A. DprE1 residual activity following a 5 min. incubation with BTZ043 or PBTZ169 (0-20 µM) in presence of 200 µM FPR, measured by a peroxidase-coupled assay, using Amplex Red as a substrate. The enzyme (5 µM) was incubated at 30 °C with inhibitor and 200 µM FPR. An aliquot was taken and diluted in the assay mixture containing 400 µM FPR, 0.2 µM horseradish peroxidase and 50

μM Amplex Red. The peroxidase activity was then assessed by continuous measurement of the fluorescence with excitation/emission wavelengths of 560/590 nm, respectively. Datapoints are the average of triplicate experiments, expressed as the percentage of the control (no inhibitor), and error bars show the observed standard error of the mean.

B. Deconvoluted mass spectrometry analysis of *M. tuberculosis* DprE1 after exposure to PBTZ169 in presence of the substrate FPR (top panel), and a protein only control (bottom panel). The protein expressed in *E. coli* presents a mass loss of 131 mass units compared to the expected mass (52326), corresponding to the excision of a methionine residue. Loss of the N-terminal methionine of proteins expressed in *E. coli* is often reported, especially when the second residue is a glycine, as here (Hirel et al., 1989). The expected masses were therefore 52195 (free protein) and 52635 (PBTZ169-DprE1 semimercaptal adduct).
