# Supplementary material for: Towards a new combination therapy for tuberculosis with next generation benzothiazinones
Source: EMBO Mol Med. 2014 Feb 5;6(3):372–83. doi: 10.1002/emmm.201303575 (PMC3958311; doi:10.1002/emmm.201303575)
Supplement: Supplementary file 4 [file emmm0006-0372-sd4.pdf]

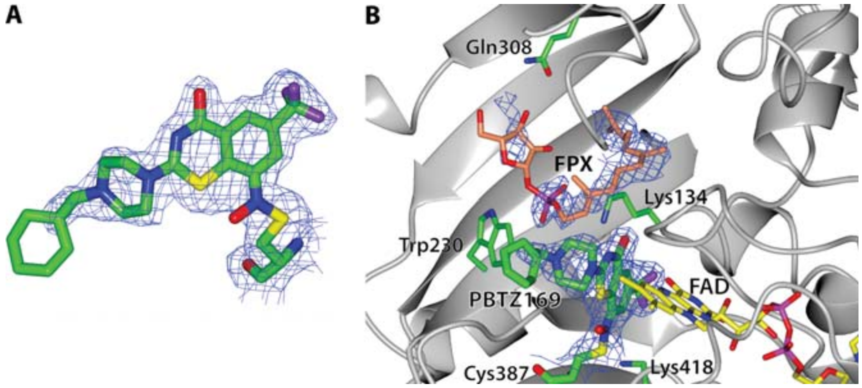

Figure S3. Structure of DprE1-PBTZ169 complex. A. Refined $2F_o$-F_c electron density map contoured at 1.0 sigma provides clear evidence of the formation of the semimercaptal adduct between the reduced form of PBTZ169 and Cys387. No electron density is observed for the cyclohexyl moiety of PBTZ169. **B.** Extra electron density is observed next to PBTZ169, which is likely due to bound FPX, the oxidation product of the FPR substrate used to prepare the DprE1-PBTZ169 complex. The electron density does not account for the sugar moiety of FPX, which was therefore not included in the final structure. Mass spectrometry analysis of dissolved crystals to determine whether FPX was indeed present, could not be performed due to the presence of PPG400 in the crystallization condition.
