# Supplementary material for: Towards a new combination therapy for tuberculosis with next generation benzothiazinones
Source: EMBO Mol Med. 2014 Feb 5;6(3):372–83. doi: 10.1002/emmm.201303575 (PMC3958311; doi:10.1002/emmm.201303575)
Supplement: Supplementary file 5 [file emmm0006-0372-sd5.pdf]

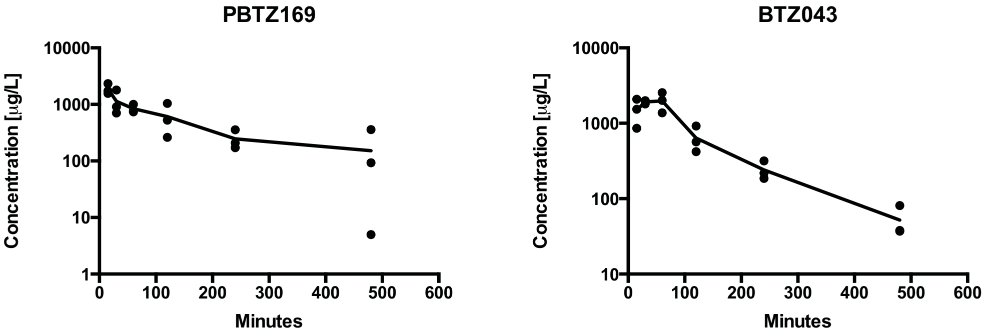

Figure S4. Comparative pharmacokinetic analysis. After receiving a single dose (25 mg/kg) of PBTZ169 or BTZ043 by gavage, groups of three female mice were sacrificed at the times indicated, blood was obtained by cardiac puncture and the drug levels in serum determined by LC-MS/MS technique (limit of quantification 10 µg/L).
