# Supplementary material for: Towards a new combination therapy for tuberculosis with next generation benzothiazinones
Source: EMBO Mol Med. 2014 Feb 5;6(3):372–83. doi: 10.1002/emmm.201303575 (PMC3958311; doi:10.1002/emmm.201303575)
Supplement: Supplementary file 6 [file emmm0006-0372-sd6.pdf]

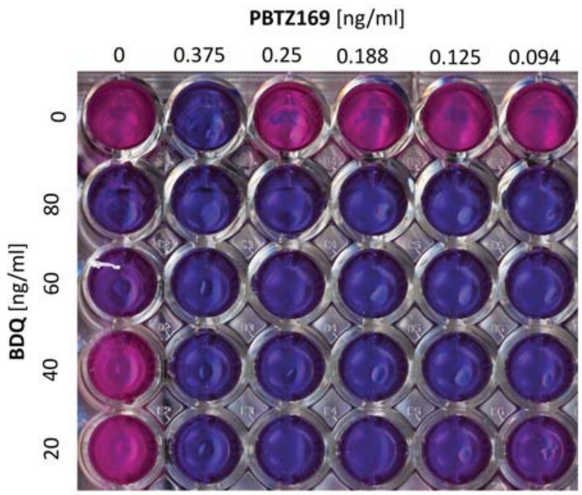

Figure S5. REMA of PBTZ169-Bedaquiline *in vitro* combinations against *M. tuberculosis* H_{37}Rv. The effect of serial dilutions of PBTZ169 and BDQ in combination on the viability of *M. tuberculosis* H37Rv was assessed after 7 days exposure. The combinations of sub-inhibitory concentrations of the two compounds are synergistic and prevent resazurin turnover.
