# Supplementary material for: Towards a new combination therapy for tuberculosis with next generation benzothiazinones
Source: EMBO Mol Med. 2014 Feb 5;6(3):372–83. doi: 10.1002/emmm.201303575 (PMC3958311; doi:10.1002/emmm.201303575)
Supplement: Supplementary file 7 [file emmm0006-0372-sd7.pdf]

Supplementary tables

Table S1. PBTZ (2-[4-R-piperazin-1-yl]-8-nitro-6-(trifluoromethyl)-4*H*-1,3-benzothiazin-4-ones) analogues synthesized and their activity on *M. tuberculosis* H37Rv

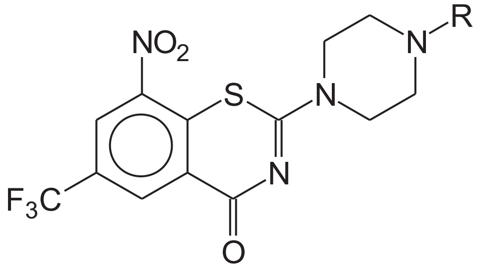

Compound	R	Molecular weight	Log*P* [a]	MIC (ng/ml)
10626038	4-chlorophenyl	470.8606	3.90	>1000
10626041	3-chloro-6-methyl-phenyl	484.8877	3.72	>1000
10626044	3-trifluoromethylphenyl	504.4140	4.27	>1000
10626045	4-fluorophenyl	454.4060	3.53	50
10626057	diethylcarboxamide	459.4504	1.44	32
10726001	4-methylphenyl	450.4427	3.85	>1000
10726002	phenyl	436.4156	3.39	25
10726004	2-fluorophenyl	454.4060	3.53	>1000
10726019	benzyl	450.4427	3.01	>500
10726040	2-phenylethyl	464.4698	3.26	5
10726044	allyl	400.3822	1.97	1500
10726045	Pyridine-2	437.4032	3.24	>1000
10726046	4-methoxyphenyl	466.4421	3.13	>1000
10726047	2-methoxyphenyl	466.4421	3.13	>1000
10726048	1,3-benzodioxol-5-yl	480.4256	3.07	>1000
10726049	3-phenylprop-2-en-1-yl	476.4809	3.67	10
**10926013**	**methyl**	**374.3439**	**1.31**	**250**
10926017	Dimethyl iodide	516.2834	0.76	>1000
10926019	1-methylpiperidine-4-yl	457.4781	2.64	>1000
**10926021**	**ethyl**	**388.3710**	**1.64**	**62**
10926023	3,4,5-trimethoxyphenyl	540.5222	2.63	250
**10926027**	**propyl**	**402.3981**	**2.11**	**3.7**
10926168	cyclohexyl	442.4634	3.09	0.75
**PBTZ169**	**cyclohexylmethyl**	**456.4905**	**3.20**	**≤0.19**

Table S1 (continued)

Compound	R	Molecular weight	Log*P* [a]	MIC (ng/ml)
10926170	1,3-dioxolan-2-ylmethyl	446.4080	1.06	31
10926171	tetrahydrofuran-2-ylmethyl	444.4357	1.51	7.5
**10926172**	**butyl**	**416.4252**	**2.51**	**1.9**
10926173	2-methoxyethyl	418.3975	2.07	31
11026088	isopropyl	402.3981	2.99	7.5
11026096	ethyl carboxylate	432.3810	2.50	31
**11026100**	**isobutyl**	**416.4252**	**2.51**	**≤1.9**
11026117	ethyl-2-morpholine	473.4775	1.78	375
11026119	N-isopropylacetylamide	459.4504	1.91	375
11026120	N,N-dimethylamine-3-prop-1-yl	445.4669	2.26	125
11026122	N,N-diethylamine-2-etan-1-yl	487.5482	2.83	1.5
11026125	2-ethanol	404.3704	0.79	750
11026126	3-propanitril	413.3809	1.41	190
11026127	N,N-dimethylamine-3-ethan-1-yl	431.4399	1.21	3100
**11026128**	**1-sec-butyl**	**416.4252**	**2.52**	**0.37**
**11026129**	**2-cyclohexylethyl**	**470.5176**	**3.52**	**0.19**
11026130	2-oxo-2-piperidin-1-ylethyl	485.4886	1.12	7.5
**11026131**	**1-methylbutyl**	**430.4523**	**3.11**	**0.19**
11026132	(1-methylpiperidin-4-yl)methyl	471.5052	1.63	3100
11026133	cyclopentyl	428.4363	2.42	1.5
**11026134**	**heptyl**	**458.5065**	**3.30**	**0.19**
11026135	pyridine-4-ylmethyl	451.4303	2.63	15
11026137	4-phenoxybutyl	508.5234	3.35	1.5
11026138	3-methoxypropyl	432.4246	2.12	3.75
11026139	4-phenylbutyl	492.5240	4.05	0.37
11026140	3-pyridin-4-ylpropyl	552.4064	3.27	3100
11026141	pentyl	430.4523	2.84	0.37
**11026142**	**1-ethylpropyl**	**430.4523**	**2.99**	**0.37**
11026167	cyclopropylmethyl	414.4092	2.94	3.75
**11026168**	**2-ethylisonicotinate**	**509.4673**	**3.09**	**500**
11027187	3-cyclohexylpropyl	484.5447	3.85	≤1.9
11026188	1-adamantyl	494.5399	2.95	15
11026189	(2-cyclohexyl-methoxy)ethyl	494.4963	2.97	19
11026191	2-(4-fluorophenoxy)ethyl	512.4867	2.99	≤1.9
11126039	benzoyl	464.4262	2.45	16

[a] Calculated using Hyperchem 7.5 (Hypercube Inc., http://www.hyper.com)
