# Supplementary material for: Towards a new combination therapy for tuberculosis with next generation benzothiazinones
Source: EMBO Mol Med. 2014 Feb 5;6(3):372–83. doi: 10.1002/emmm.201303575 (PMC3958311; doi:10.1002/emmm.201303575)
Supplement: Supplementary file 8 [file emmm0006-0372-sd8.pdf]

Table S2. Susceptibility of clinical isolates to PBTZ169 and INH

Strain/clinical isolates of *M. tuberculosis*	Resistance to	Type	MIC (μg/mL) PBTZ169	MIC (μg/mL) INH
H37Rv	-	Sensitive	0.031	0.125
4328	-	Sensitive	0.063	0.125
6341	HSRE	MDR	0.031	>20
6374	HSRE	MDR	0.063	>20
5055	HSRE	MDR	0.063	>20
5253	HSREKmRbZ	pre-XDR	0.063	>20
6412	HSRE	MDR	0.031	>20
6448	HSRE	MDR	0.063	>20
6418	HSREKmFQ	XDR	0.063	>20
7384	HSREKmFQ	XDR	0.063	>20

Strains 4328, 6341, 6374,5055, 5253, 6412, 6448, 6418, and 7384 are clinical isolates from patients of the Central Institute of Tuberculosis (Moscow. Russia) where drug susceptibility testing was performed using solid Löwenstein-Jensen medium. The results were read after 21-28 days of incubation at 37°C. Controls were tubes cultured with test-strains not treated with the studied agents. Drugs: H, isoniazid (INH); S, streptomycin; R, rifampicin; E, ethambutol; Km, kanamycin; Rb, rifabutin; Z, pyrazinamide; FQ, fluoroquinolone. Note that the MICs for PBTZ169 are higher than in Table 2 in the main text due to the use of solid medium.
