# Supplementary material for: Towards a new combination therapy for tuberculosis with next generation benzothiazinones
Source: EMBO Mol Med. 2014 Feb 5;6(3):372–83. doi: 10.1002/emmm.201303575 (PMC3958311; doi:10.1002/emmm.201303575)
Supplement: Supplementary file 9 [file emmm0006-0372-sd9.pdf]

Table S3. Data collection and refinement statistics for the DprE1-PBTZ169 structure

	**DprE1-PBTZ169**
**Data collection**	
Space group	$P2_1$
Cell dimensions	
a, b, c (Å)	73.05, 85.00, 81.37
α, β, γ (°)	90.00, 101.10, 90.00
Resolution (Å)	50.0-1.9
R_{meas}	6.6 (52.1)
$I / \sigma I$	10.4 (2.1)
Completeness (%)	95.3 (84.9)
Redundancy	2.3 (2.3)
**Refinement**	
Resolution (Å)	1.90
R_{work} / R_{free}	0.19/0.24
No. atoms	
Protein	6456
PBTZ/FAD	60/106
Water	255
B-factor (A^2)	38.2
R.m.s deviations	
Bond lengths (Å)	0.022
Bond angles (°)	2.20

1 crystal used for the structure.
*Highest resolution shell is shown in parenthesis.
