# Supplementary material for: Towards a new combination therapy for tuberculosis with next generation benzothiazinones
Source: EMBO Mol Med. 2014 Feb 5;6(3):372–83. doi: 10.1002/emmm.201303575 (PMC3958311; doi:10.1002/emmm.201303575)
Supplement: Supplementary file 10 [file emmm0006-0372-sd10.pdf]

**Table S4. Selected *in vitro* ADME/T values**

**Cytotoxicity**

BTZ043 and PBTZ169 were tested for cytotoxicity against HepG2 by REMA in triplicate. Toxic doses inhibiting 99% and 50% of the population are expressed as $TD_{99}$ and $TD_{50}$. BTZ043 is around 10 fold more cytotoxic than PBTZ169 for HepG2 cells

| | $TD_{99}$ (µg/ml) | $TD_{50}$ (µg/ml) |
|---|---|---|
| **BTZ043** | 25 | 5 |
| **PBTZ169** | >200 | 58 |

**Microsomal stability**

Intrinsic clearance (Clint: measured in µl/min/mg protein) was measured using both mouse and human liver microsomes. Compound concentrations were measured by HPLC-UV following 0, 5, 10, 15 and 30 min incubation of the compound with microsomes. Carbamazepine acts as a control for low intrinsic clearance, while nifedipine is a control for high intrinsic clearance. BTZ043 and PBTZ169 both show intermediate intrinsic clearance.

| **Microsomes** | **1 µg/mL Carbamazepine** | **1 µg/mL Nifedipine** | **1 µM BTZ043** | **1 µM PBTZ169** |
|---|---|---|---|---|
| **Mouse** | 1.1 | 64.8 | 12.2 | 24.3 |
| **Human** | 0.5 | 72.3 | 16.0 | 24.8 |
