# Supplementary material for: Towards a new combination therapy for tuberculosis with next generation benzothiazinones
Source: EMBO Mol Med. 2014 Feb 5;6(3):372–83. doi: 10.1002/emmm.201303575 (PMC3958311; doi:10.1002/emmm.201303575)
Supplement: Supplementary file 11 [file emmm0006-0372-sd11.pdf]

Table S5. Principal pharmacokinetic properties[1]

		BTZ043	PBTZ169
C_{max}	[µg/L]	1922.9	1849.1
λ_z	[min-1]	0.00694	0.00623
$t1/2_z$	[min]	99.9	111.3
AUC	[min·µg/L]	259829	193487

[1] See Figure S5 for primary data. Non-compartmental calculations gave these parameter estimates. Compound levels were measured in serum from individual female mice (3/time point) that had received a single dose of 25 mg/kg. Maximum concentration (C_{max}); area under the curve (AUC); apparent clearance.
