# Supplementary material for: Towards a new combination therapy for tuberculosis with next generation benzothiazinones
Source: EMBO Mol Med. 2014 Feb 5;6(3):372–83. doi: 10.1002/emmm.201303575 (PMC3958311; doi:10.1002/emmm.201303575)
Supplement: Supplementary file 12 [file emmm0006-0372-sd12.pdf]

Table S6. Statistical comparison of *in vivo* combination therapies[1]

Log$_{10}$ CFU reduction compared to standard combination RIF-INH-PZA

**Lungs**	p value (t test)	Signif.different $p < 0.05$	**Spleen**	p value (t test)	Signif.different $p < 0.05$
**After 4 weeks**			**After 4 weeks**		
PBTZ+PZA	0.002	Yes	PBTZ+PZA	0.571	No
PBTZ+BDQ	0.004	Yes	PBTZ+BDQ	0.002	Yes
BDQ + PZA	0.001	Yes	BDQ + PZA	0.009	Yes
PBTZ+BDQ+PZA	N/A	N/A	PBTZ+BDQ+PZA	0.003	Yes
**After 8 weeks**			**After 8 weeks**		
PBTZ+PZA	0.619	No	PBTZ+PZA	0.230	No
PBTZ+BDQ	0.251	No	PBTZ+BDQ	0.017	Yes
BDQ + PZA	0.144	No	BDQ+PZA	0.077	No
PBTZ+BDQ+PZA	0.046	Yes	PBTZ+BDQ+PZA	0.015	Yes

[1] See Figure 9 for primary data
