# Supplementary material for: Towards a new combination therapy for tuberculosis with next generation benzothiazinones
Source: EMBO Mol Med. 2014 Feb 5;6(3):372–83. doi: 10.1002/emmm.201303575 (PMC3958311; doi:10.1002/emmm.201303575)
Supplement: Supplementary file 13 [file emmm0006-0372-sd13.pdf]

Supporting Information

Towards a new combination therapy for tuberculosis with next generation benzothiazinones

Vadim Makarov[1,2,†], Benoit Lechartier[1,3,†], Ming Zhang[1,3,†], João Neres[1,3,4†], Astrid M. van der Sar[5], Susanne A. Raadsen[5,°], Ruben C. Hartkoorn[1,3], Olga B. Ryabova[1,2], Anthony Vocat[1,3], Laurent Decosterd[6], Nicolas Widmer[6], Thierry Buclin[6], Wilbert Bitter[4,7], Koen Andries[8], Florence Pojer[1,3], Paul J. Dyson[4] & Stewart T. Cole[1,3,*]

TABLE OF CONTENTS

Synthetic route for PBTZ169......2

DprE1 purification and crystallography procedures......4

References......5

Table S1. PBTZ (2-[4-R-piperazin-1-yl]-8-nitro-6-(trifluoromethyl)-4*H*-1,3-benzothiazin-4-ones) analogues synthesized and their activity on *M. tuberculosis*H37Rv......8

Table S2. Susceptibility of clinical isolates to PBTZ169 and INH......9

Table S3. Data collection and refinement statistics for the DprE1-PBTZ169 structure. 10

Table S4. Selected *in vitro* ADME/T values. 11

Table S5. Principal pharmacokinetic properties 12

Table S6. Statistical comparison of *in vivo* combination therapies......13

Figure S1. Susceptibility of BTZ-resistant mutants of *M. smegmatis* to PBTZ169.......14

Figure S2. Enzyme inhibition and mass spectrometry analysis of the covalent DprE1-inhibitor adducts.......15

Figure S3. Structure of DprE1-PBTZ169 complex. 17

Figure S4. Comparative pharmacokinetic analysis. 18

Figure S5. REMA analysis of synergistic combinations......19

Synthetic route for PBTZ169

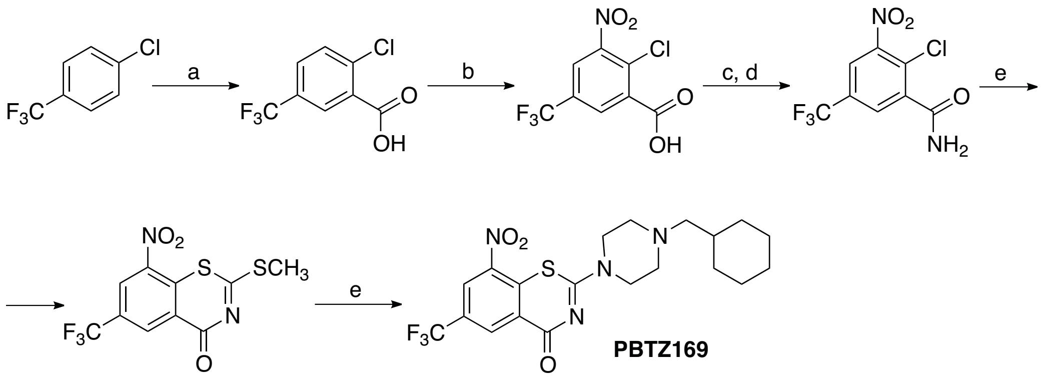

a) At –55-65°C, butyllitium (200 mL, 1.6 M solution in hexane) was added to a solution of 4-chlorobenzotrifluoride (39 mL) and TMEDA (45 mL) in 500 mL of THF. The mixture was stirred for 1 h at –55-65° C, and then poured onto 500 g freshly crushed dry ice. The solvents were evaporated and the residue dissolved in water (about 200 mL), washed with diethyl ether (3 x 50 mL) and acidified with conc. HCl to pH 1. Extraction with dichloromethane (3 x 100 ml) followed by crystallization from hexane gave **2-chloro-5-(trifluoromethyl)benzoic acid** as a white solid (m.p. 93-94°C).

b) To a mixture of 250 ml of conc. H_2SO_4 (98%) and 36 ml of fuming HNO_3 (95-98%) was added 72 g of dry 2-chloro-5-trifluoromethylbenzoic acid in small portions, at room temperature with stirring. After 30 minutes the reaction mixture was slowly heated to 90°C. During this process a white solid formed and the reaction mixture became dense. Addition of conc. H_2SO_4 (up to 100 mL) helps solubility of the reaction mixture, which was stirred for 40 minutes, cooled to room temperature and mixed with 2 kg of fine ice. The resultant white solid was filtered off and washed with cold water. Recrystallization of the solid from a water/ethanol mixture gave **2-chloro-3-nitro-5-(trifluoromethyl)benzoic acid** as a white solid (m.p. 188-190°C).

c) A suspension of 32.0 g of 2-chloro-3-nitro-5-(trifluoromethyl)benzoic acid in 200 ml CCl_4, 1,2 mL of dry DMF and 20 mL of $SOCl_2$ was refluxed during 3 hours, when the reaction mixture was a clear solution. The solvent was removed under vacuum

affording **2-chloro-3-nitro-5-(trifluoromethyl)benzoyl chloride** as an oil, which was used in next step without any purification.

d) A solution of 50 g of 2-chloro-3-nitro-5-(trifluoromethyl)benzoyl chloride in 100 mL of acetonitrile was slowly added to 600 mL of 12.5% ammonia solution in water at 0 - –10°C. After 7 min. the reaction mixture was poured onto 500 mL of cold water and the white precipitate recovered by filtration. Recrystallization from a water/ethanol mixture afforded **2-chloro-3-nitro-5-(trifluoromethyl)benzamide** as a white solid (m.p. 200-203°C).

e) 2-Chloro-3-nitro-5-(trifluoromethyl)benzamide (20g) was added to a solution of sodium hydroxide (5,9 g) and carbon disulfide (13,5 mL) in 70 mL of DMSO, below 10°C. After 15 minutes, 5 mL of MeI was slow added and solid formation was observed. The reaction mixture was stirred for 30 min and water was added, keeping the temperature below 20°C, with an ice/water bath. The yellow solid thus formed was filtered off and recrystallized from a small volume of ethyl acetate, affording . **2-(methylthio)-8-nitro-6-(trifluoromethyl)-4H-thiochromen-4-one** as a pale yellow solid (m.p 200-3°C, 11 g).

f) To a suspension of of 2-(methylthio)-8-nitro-6-(trifluoromethyl)-4H-thiochromen-4-one (3 g) in 15 mL of ethanol was added 4-cyclohexylmethylpiperazine {1.8 mL; d = 0.938 - J.Med.Chem. 35 (1992), 14, 2688)}, with stirring, at room temperature. The reaction mixture was heated to 50-60°C under continuous stirring for 20 minutes. After cooling, 100 mL of water was added and the resulting light yellow solid was recovered by filtration, and dried. Recrystallization from ethanol afforded **2-[4-(cyclohexylmethyl)piperazin-1-yl]-8-nitro-6-(trifluoromethyl)-4H-1,3-benzothiazin-4-one (PBTZ169)** as a yellow solid (m.p. 184-186°C):Yield: 71%; HRMS (ESI$^+$) calcd for $H_{24}F_3N_4O_3S$ $[M + H]^+$: 457.1516; found: 457.1525 (error 2.0 ppm). ^{1}H NMR (300 MHz, DMSO-d_6) δ 8.86 (s, 1H, CH_{ar}), 8.76 (s, 1H, CH_{ar}), 3.91 (broad s, 4H, $N(CH_2)_2$), 2.51 (broad s, 4H, $N(CH_2)_2$), 2.13 (d, J = 7.4 Hz, 2H, CH_2), 1.80-1.60 (m, 5H, $5CH_{cyclohexyl}$), 1.56-1.46 (m, 1H, CH), 1.30-1.14 (m, 3H, $3CH_{cyclohexyl}$), 0.91-0.79 (m, 2H, $2CH_{cyclohexyl}$) ppm.

DprE1 purification and crystallography procedures

The *M. tuberculosis dprE1* gene was cloned into a pET28a plasmid and co-expressed with *M. tuberculosis* GroEL2 (Rv0440) in *E. coli* GroES, BL21 (DE3) cells. For this purpose, the pGro7 plasmid (Takara Bio Inc.) was modified to replace the *E. coli* GroEL gene by the *M. tuberculosis groEL2* (Rv0440) gene. Cells were transformed with both plasmids and grown in auto-inducing medium (AIM LB Broth base including trace elements, Formedium, U.K.) containing 250 µg/ml L-arabinose, 50 µg/ml kanamycin and 20 µg/ml chloramphenicol for 3h at 37 °C and then overnight at 16 °C. Cells were harvested by centrifugation and resuspended in 50 mM Tris-HCl pH 8.0, 500 mM NaCl, 5% (v/v) glycerol, 1% CHAPS, 1 mM phenylmethanesulfonylfluoride (20 g of cells/100 mL buffer containing 2 tablets of complete EDTA-free protease inhibitors cocktail, Roche). Cell disruption was carried out by sonication and the clear lysate was obtained by centrifugation at 18,000 rpm for 1 h at 4 °C. Protein purification was carried out on an AktaPurifier FPLC system. The clear lysate was loaded onto a 5 mL HisTrap HP column (GE Healthcare), followed by elution of unspecifically-bound proteins with 50 mM imidazole. The protein was eluted with a gradient of increasing imidazole concentration, the fractions containing DprE1 were pooled and concentrated on an Amicon centrifugal device (30,000 MWCO, Millipore), and the buffer exchanged to 50 mM Tris-HCl pH 8.5. The protein was loaded on a MonoQ 5/50 GL column (GE Healthcare), washed with 50 mM Tris-HCl pH 8.5 and then eluted with a 0-500 mM NaCl gradient in 20 min. Fractions containing pure protein were pooled, concentrated and flash-frozen in liquid nitrogen for storage at -80 °C.

For crystallization purposes, *M. tuberculosis* DprE1 (approximately 20 µM) was incubated for 3h at 30 °C, 100 µM PBTZ169 and 200 µM FPR, in 20 mM Tris pH 8.5, 100 mM NaCl. The protein was concentrated to approximately 7 mg/mL on an Amicon centrifugal device (30,000 MWCO, Millipore). Crystals were obtained by the hanging-drop vapor diffusion method at 18 °C. Experiments were set up by mixing 1 µl of the protein sample with 1 µl of the reservoir solution containing 100 mM imidazole, pH 7.2, 36% polypropyleneglycol 400. Yellow crystals grew in approximately 1-3 days and were transferred to a cryo-protectant (reservoir solution with 25% glycerol) prior to flash-cooling in liquid nitrogen.

X-ray diffraction datasets were collected at the X06DA beamline of the Swiss Light Synchrotron (SLS, Villigen). Data processing and scaling were carried out in XDS (Kabsch, 2010) (**Table S3**). The DprE1-PBTZ169 complex crystal structure (PDB 4NCR) was solved by molecular replacement using the native DprE1 structure (PDB 4FDP) as model. Manual adjustments of the model were made in COOT (Emsley and Cowtan, 2004), including the addition of PBTZ169, waters, glycerol and imidazole, followed by refinement using REFMAC5 (Murshudov et al., 1997), part of the CCP4i program suite (Winn et al., 2011). In the refined structures all residues fall in the allowed regions of the Ramachandran plot.

References

Emsley, P., Cowtan, K., 2004. Coot: model-building tools for molecular graphics. Acta Crystallographica Section D-Biological Crystallography 60, 2126-2132.

Hirel, P.H., Schmitter, M.J., Dessen, P., Fayat, G., Blanquet, S., 1989. Extent of N-terminal methionine excision from Escherichia coli proteins is governed by the side-chain length of the penultimate amino acid. Proc Natl Acad Sci U S A 86, 8247-8251.

Kabsch, W., 2010. XDS. Acta Crystallographica Section D-Biological Crystallography 66, 125-132.

Makarov, V., Manina, G., Mikusova, K., Mollmann, U., Ryabova, O., Saint-Joanis, B., Dhar, N., Pasca, M.R., Buroni, S., Lucarelli, A.P., Milano, A., De Rossi, E., Belanova, M., Bobovska, A., Dianiskova, P., Kordulakova, J., Sala, C., Fullam, E., Schneider, P., McKinney, J.D., Brodin, P., Christophe, T., Waddell, S., Butcher, P., Albrethsen, J., Rosenkrands, I., Brosch, R., Nandi, V., Bharath, S., Gaonkar, S., Shandil, R.K., Balasubramanian, V., Balganesh, T., Tyagi, S., Grosset, J., Riccardi, G., Cole, S.T., 2009. Benzothiazinones kill Mycobacterium tuberculosis by blocking arabinan synthesis. Science 324, 801-804.

Murshudov, G.N., Vagin, A.A., Dodson, E.J., 1997. Refinement of macromolecular structures by the maximum-likelihood method. Acta Crystallographica Section D-Biological Crystallography 53, 240-255.

Winn, M.D., Ballard, C.C., Cowtan, K.D., Dodson, E.J., Emsley, P., Evans, P.R., Keegan, R.M., Krissinel, E.B., Leslie, A.G.W., McCoy, A., McNicholas, S.J., Murshudov, G.N., Pannu, N.S., Potterton, E.A., Powell, H.R., Read, R.J., Vagin,

A., Wilson, K.S., 2011. Overview of the CCP4 suite and current developments. Acta Crystallographica Section D-Biological Crystallography 67, 235-242.
